# Contactless Vital Signs Monitoring From Videos Recorded With Digital Cameras: An Overview

**DOI:** 10.3389/fphys.2022.801709

**Published:** 2022-02-18

**Authors:** Nunzia Molinaro, Emiliano Schena, Sergio Silvestri, Fabrizio Bonotti, Damiano Aguzzi, Erika Viola, Fabio Buccolini, Carlo Massaroni

**Affiliations:** ^1^Unit of Measurements and Biomedical Instrumentation, Departmental Faculty of Engineering, Università Campus Bio-Medico di Roma, Rome, Italy; ^2^BHOHB – Biometrical Holistic of Human Body S.r.l., Rome, Italy

**Keywords:** contactless monitoring, remote vital signs monitoring, digital camera, remote photoplethysmography, respiratory rate, pulse rate, blood pressure, SpO_2_

## Abstract

The measurement of physiological parameters is fundamental to assess the health status of an individual. The contactless monitoring of vital signs may provide benefits in various fields of application, from healthcare and clinical setting to occupational and sports scenarios. Recent research has been focused on the potentiality of camera-based systems working in the visible range (380–750 nm) for estimating vital signs by capturing subtle color changes or motions caused by physiological activities but invisible to human eyes. These quantities are typically extracted from videos framing some exposed body areas (e.g., face, torso, and hands) with adequate post-processing algorithms. In this review, we provided an overview of the physiological and technical aspects behind the estimation of vital signs like respiratory rate, heart rate, blood oxygen saturation, and blood pressure from digital images as well as the potential fields of application of these technologies. Per each vital sign, we provided the rationale for the measurement, a classification of the different techniques implemented for post-processing the original videos, and the main results obtained during various applications or in validation studies. The available evidence supports the premise of digital cameras as an unobtrusive and easy-to-use technology for physiological signs monitoring. Further research is needed to promote the advancements of the technology, allowing its application in a wide range of population and everyday life, fostering a biometrical holistic of the human body (BHOHB) approach.

## Introduction

The measurement of physiological parameters is pivotal to evaluate the health status of an individual quantitatively. Among the wide range of measurable physiological data, the five vital signs routinely monitored in the clinical practice are the respiratory frequency or breathing rate (*f_R_*), the pulse or heart rate (HR), the blood pressure (BP), the blood oxygen saturation (SpO_2_) and the body temperature (BT; Elliott and Coventry, 2012).

Typically, physiological parameters are measured or estimated using contact-based devices that require direct contact with the skin and human body. Moreover, the different vital signs are usually retrieved from raw signals recorded in various body landmarks (e.g., *f_R_* with flow sensors at the level of the mouth (Schena et al., 2015), HR from the electrocardiogram signal recorded *via* skin-attached electrodes (Kranjec et al., 2014), SpO_2_ from the blood volume variations at the level of the finger with the photoplethysmographic (PPG) sensors (Yoon et al., 2002), and BP with a cuff-based measuring system at the level of the upper arm (Chung et al., 2013). However, these contact-based techniques may have some drawbacks, such as loss of contact, skin irritations, or damage in subjects with vulnerable and fragile skin (Harford et al., 2019; Paul et al., 2020), may compromise the reliability of measurements, and in some cases, they may not be employable for long duration and remote continuous monitoring (Kebe et al., 2020).

To overcome these issues, non-contact techniques for estimating one or more vital signs even remotely are gaining much interest (Massaroni et al., 2019b, 2021). Numerous are the fields of application in which these technologies can be suitable and advantageously employed, from healthcare and telemedicine (Zhao et al., 2013) to occupational settings (Massaroni et al., 2019a), sport science (Nicolò et al., 2020), automotive field (Zhang et al., 2017), emotion recognition (Grassmann et al., 2016; McDuff et al., 2016), and in everyday situations where unobtrusiveness and ease to use can be favorable without neglecting an adequate level of measurement’s reliability and accuracy (Hall et al., 2017). Contactless technologies for physiological monitoring are numerous and based on heterogeneous operating principles, with different levels of non-invasiveness required to capture the signals and users’ comfort (Sun, 2011; Tarassenko et al., 2014; Zhang et al., 2017; Massaroni et al., 2019a; Rossol et al., 2020).

Besides the plethora of available technologies, some promising technologies are based on retrieving valuable signals from the external surface of the body to estimate the values of *f_R_*, HR, SpO_2_, and BP, as Doppler radar (Boric-Lubecke et al., 2009), optical vibrocardiography (Morbiducci et al., 2007), thermal imaging (Garbey et al., 2004), Laser Doppler Vibrometry (LVD; Antognoli et al., 2020). Such techniques allow the contactless measurement of the physiological parameters without interfering with the comfort of the subject, facilitating the monitoring of physiological signs even in challenging conditions (e.g., ambient lighting and different skin tones; Hassan et al., 2017b; Shirbani et al., 2020). The only disadvantage of such techniques is the need for dedicated hardware and complex setups for measurement.

A simple video captured with a digital camera working in visible light wavelength range (approximately 380–750 nm; i.e., the optical sensors embedded in smartphones, modern security surveillance cameras, laptop webcams) can be used to retrieve physiological-related signals from different body areas (Tarassenko et al., 2014; Massaroni et al., 2018b; Antognoli et al., 2019; Rossol et al., 2020). The face and the torso provide a good trade-off between unobtrusiveness and sensitivity to cardiorespiratory changes, thus enabling the monitoring of all the other vital signs even in daily-life conditions.

The use of cameras in the visible range is attracting increasing attention within the scientific community for a wide range of healthcare applications and services. Systematic reviews exclusively focused on digital image processing techniques for the estimation of various physiological parameters (e.g., Khanam et al., 2019), as well as on specific measurement techniques (e.g., remote photoplethysmography) to estimate a limited number of parameters (Sun and Thakor, 2016; Zaunseder et al., 2018) are already present in literature. Different from what is already available, this review aims to provide a complete overview of possible use of digital cameras working in visible light wavelength range to monitor all the vital signs, specifying guidelines for using this technology and the raw signals in the different scenarios (from clinical to sports science settings) and providing an overview of current research evidence, gaps and potential developments in the field. This review investigates principles, methods and applications which were published in international journals and conferences from 2011 until 2021. Papers were searched in Digital Library and Bibliographic Search databases (e.g., Google Scholar, SCOPUS, and PubMed) with the following terms combinations: “remote,” “contactless,” “non-contact,” “video,” “camera-based,” “vital signs,” “physiological parameters,” “cardio-respiratory,” “respiratory,” “breathing,” “heart rate,” “pulse rate,” “photoplethysmography,” “SpO_2_,” “blood oxygen saturation,” “blood pressure,” “body temperature,” “skin temperature” in titles and abstracts. To be focused on the specific technology and its fields of application, studies using NIR/IR cameras, depth cameras, multi-camera systems working in the IR wavelength range—typically used for motion capture—were not included in the present review.

The review is structured in the following sections. The section *Cardiorespiratory physiology* provides basic introductory principles on body movements and vascularization of the face and hands to understand the physiological basis underneath the non-contact physiological monitoring through visible light. In the section *Measurement of physiological parameters from visible light-exposed surface of the body,* the main methods used to extract the different parameters are described. Then the section *Monitoring of physiological parameters and possible fields of application* summarizes the most important studies that use digital cameras to estimate the main vital signs (i.e., *f_R_*, HR, SpO_2_, BP) in different scenarios. A brief introduction on the importance of monitoring in different scenarios is provided for each parameter, along with the advantages of using remote monitoring in these settings. Finally, the last section is dedicated to *Conclusions*, where problems, current research gaps, potential developments in the field are reported.

## Cardiorespiratory Physiology

The measurement of physiological parameters from external surface of the body is mainly related to the cardiorespiratory activity and the characteristics of both the respiratory and cardiovascular systems.

**Figure 1:**
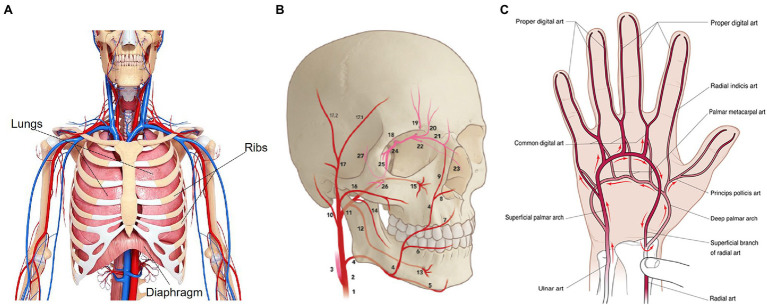
Anatomy of the respiratory system **(A)**, face (**B**; adapted from von Arx et al., 2018), and hand **(C)** vascularization (from Habib et al., 2012). In **(B)**, numbers indicate the main arteries that carry the blood to the forehead and cheeks, specifically: common carotid artery (1), external carotid artery (2), internal carotid artery (3), facial artery (4), transverse facial artery (16), superficial temporal artery (17) and frontal branch (17.1), ophthalmic artery (18).

The respiratory system is composed primarily of the chest wall, lungs, and diaphragm (Figure 1A). When dealing with the need for external recording of breathing-related movements, the chest wall is surely the best measurement site. In terms of the mechanics of pulmonary ventilation, the lungs can be expanded and contracted in two manners: (i) through the downward and upward movement of the diaphragm allowing the extension and the contraction of the chest cavity; (ii) through the elevation and depression of the ribs to increase and decrease the diameter of the chest cavity. Normal quiet breathing is performed entirely by the movement of the diaphragm: during inspiration, the lungs are pulled downward by the contraction of the diaphragm, then during expiration, the diaphragm relaxes, and the compression of the lungs and the consequent exhalation of the air is facilitated by the elastic recoil of the lungs, chest wall, and abdominal structures. The raising of the rib cage allows the expansion of the lungs since, in the natural resting position, the ribs incline downward, allowing the sternum to move backward toward the vertebral column. When the rib cage is elevated, the ribs are directly forward; thus, the sternum moves forward away from the vertebral column, making the anteroposterior thickness of the chest greater during the maximum inspiration than during expiration (Hall and Guyton, 2011).

The cardiovascular system aims to transport the blood throughout the body, and it is composed of the heart, which pumps blood, and the circulatory system. The circulatory system comprises arteries, capillaries, and veins, which contribute to maintaining the body’s tissues in healthy conditions. In this case, the face and upper extremities like hands are surely of interest for measuring cardiovascular-related phenomena with digital cameras. The systemic circulation carries the oxygenated blood from the heart to the rest of the body and transports the deoxygenated blood back to the heart to begin the process again (Al-Naji et al., 2017). Throughout the systemic circulation, there are some physical changes of vascularity (e.g., skin blanching, ecchymosis, hematoma, and edema) that are mainly visible in the face and in the palm’s hand, which are the most exposed part of the body. The supply arteries of the face are originated from the bilateral common carotid artery that starts on the right side from the brachiocephalic artery and the left side from the aorta. The common carotid artery divides into internal and external carotid arteries at the fourth cervical vertebral body level. The main arteries of the face arise either from the superficial carotid artery or from branches of the external carotid artery. Still, the major contributor to the forehead is the ophthalmic artery (i.e., artery of the orbit) originating from the internal carotid artery. Focusing on the arterial vascularization of the forehead, the frontal branch of the superficial temporal artery, with a mean diameter of about 2 mm, is the largest supplier compared to the other arteries. Instead, the main blood supply for the cheeks is from arterial perforators that originate from the transverse facial artery and the facial artery (von Arx et al., 2018; Figure 1B). The main arteries bringing oxygenated blood to the hand are the radial and ulnar arteries, the brachial artery’s terminal division. The hand has numerous networks of vessels to guarantee a proper blood flow (Nguyen and Duong, 2020). Concerning the palm’s hand, the vascular supply is derived from the superficial and the deep palmar arches (Habib et al., 2012). The superficial palmar arch is a progression of the ulnar artery and lies superficial to the flexor tendons, while the deep palmar arch, lying in the flexor tendons, is originated from the dorsal branch of the radial artery (Tan and Lahiri, 2020). The princeps pollicis artery, which supplies the thumb and the branches that communicate with the common digital arteries are originated from the deep palmar arch (Tan and Lahiri, 2020; Figure 1C).

## Measurement of Physiological Parameters From Visible Light-Exposed Surface of the Body

The physiological and physical effects deriving from the cardiorespiratory activity are essential for measuring and monitoring physiological parameters from the external surface of the body without contact. It is worth clarifying that the available scientific literature does not provide sufficient evidence about the estimation of BT from the post-processing of signals recorded with digital cameras in the visible light range (Cheng et al., 2017). For this reason, the use of cameras for BT measurement will not further explored in this review.

Although the existing methods for the post-processing of the video images may be different depending on the different parameter of interest (*f_R_*, HR, SpO_2_, BP), all can be classified as either color intensity-based or motion-based methods.

Color-based methods rely on the detection of subtle skin color changes due to the cyclical movement of the blood. The signal associated with these changes is a plethysmographic (i.e., PPG) signal, which is typically called remote-PPG (sometimes reported as rPPG) or imaging-PPG (i-PPG in short). rPPG is based on the principle that blood absorbs light more than the surrounding tissue, so blood volume variations affect light transmission and reflectance (Verkruysse et al., 2008). The estimation of cardiac-related parameters depends on the acquisition of a rPPG signal, which is obtained by analyzing the intensity changes of the pixels in the green color channel since the hemoglobin has high ability of absorption in this channel (Davila et al., 2017). When the HR is estimated from rPPG it is typically termed as pulse rate (i.e., PR). Nevertheless, we prefer to use the term HR from here on out to avoid misunderstanding in the reading. Even *f_R_* can be estimated from the rPPG signal, as the respiratory activity modulates the cardiac activity (Bernardi et al., 2001). With respect to HR, the measurement of SpO_2_ requires light at two different wavelengths. SpO_2_ is defined as the ratio between the oxygenated hemoglobin (HbO_2_) and the total amount of hemoglobin (i.e., deoxygenated and oxygenated hemoglobin), which can be optically distinguished by the different absorption of light at two different wavelengths [i.e., oxyhemoglobin has high absorption at infrared light (IR) and deoxyhemoglobin has a higher absorption at red light; Foo et al., 2013]. Considering the rPPG signal, the intensity changes of the pixels in the red color channel and in the blue color channel can be used to compute the ratio of the absorbances at the two wavelengths (de Fatima Galvao Rosa and Betini, 2020) as they correspond to the red light (i.e., *λ*_1_ = 660 nm) and the IR light (i.e., *λ*_2_ = 940 nm), respectively.

Motion-based methods are those based on the detection of small-amplitude movements recorded by a video camera (Al-Naji et al., 2017). The monitoring of respiratory-related parameters relies on the detection of the thorax movements caused by the breathing activity. Since the movement of the recorded surface (i.e., chest wall movements due to the respiratory activity) affects the changes of intensity of the pixels, the resulting changes of the reflected light intensity may be used to collect breathing patterns and related parameters indirectly (Massaroni et al., 2018b, 2019a). Another approach is related to the detection of optical flow which can be used to detect the chest surface movement (Ganfure, 2019; Massaroni et al., 2019c). Optical flow allows computing the displacement between two consecutive images by tracking the features of the images (Mateu-Mateus et al., 2020). For the estimation of cardiac-related parameters, a motion-based method is based on the detection of the head motions due to the movement of the blood from the heart to the head (sometimes known as ballistocardiography, BCG). Feature tracking can be used to extract the motion of the head, and the vertical direction is the best axis to measure the upright movement of the head (Balakrishnan et al., 2013).

When the visible light is used, a digital camera is needed to implement a video camera imaging measuring technique. The working principle of this technique is based on the use of an optical sensor that records a video of the face, palm’s hand, and/or chest of a subject. When a surface is recorded by a video, the pixel of each video’s frame has an intensity level due to the light reflected by the surface over a two-dimensional grid of pixels. These intensity levels represent a digital image that is obtained through optical sensors, like Charge Coupled Device (CCD) or Complementary Metal-Oxide Semiconductor (CMOS) sensors, which convert the light radiations into electronic signals.

**Figure 2:**
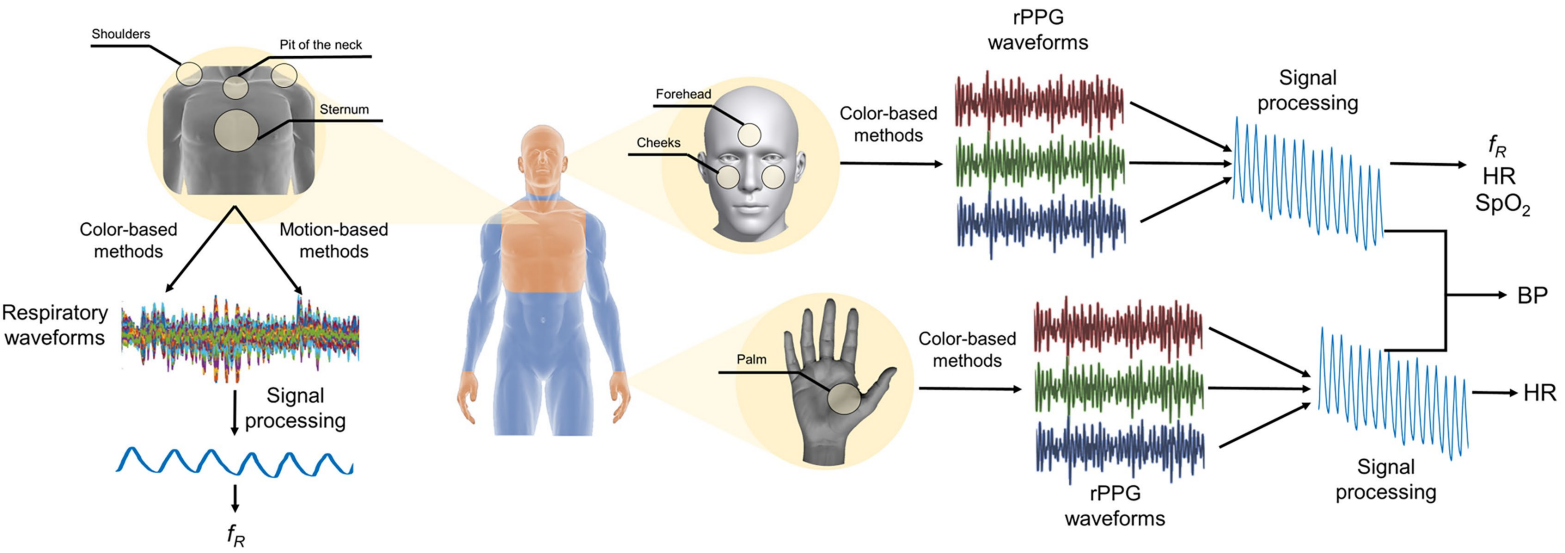
Measurement sites of the body and principal steps for extracting *f_R_*, HR, SpO_2_, and BP.

In Figure 2 is reported a schematic representation of the main measurement sites of the body and the main steps to retrieve the valuable signal for the estimation of the vital signs.

## Monitoring of Physiological Parameters and Possible Fields of Application

### Respiratory Frequency and Other Respiratory Parameters

The measurement of respiratory parameters mainly relies on chest wall movements, which are affected by both the cardiovascular and respiratory activities. During the inspiration and expiration, the chest wall moves to allow the expansion and contraction of the lungs. The chest wall is considered as a structure comprising two compartments, the rib cage, and the abdomen, that experience different displacements during respiratory activity. During the inspiration, the rib cage mainly moves in the ventral and cranial directions with a displacement in the range of 3–5 mm (Massaroni et al., 2017). Lateral movements outward are very small, between 1 and 2 mm, that are useful for the elevation of the ribs (de Groote et al., 1997). The abdomen mainly moves in the ventral direction so that it becomes circle during inspiration. At the end of the expiration, the ratio between the dorsoventral and transverse diameters is smaller for the rib cage than for the abdomen. Indeed, the abdominal cross section is near a circle than the rib cage cross section (de Groote et al., 1997). By recording these cyclical movements through a digital camera, a respiratory pattern can be obtained by analyzing the changes in the intensity level of the pixels of each frame of the video (Massaroni et al., 2019a). Moreover, the respiratory activity has a modulatory effect on the cardiac activity (Bernardi et al., 2001): when a person inhales, the HR tends to increase, whereas when a person exhales, the HR tends to fall. That is the reason why the *f_R_* can be extracted from rPPG signals (Chen et al., 2019b). Furthermore, as reported in (van Gastel et al., 2016), the respiration modulates the rPPG waveform in three ways: (i) respiratory induced frequency variation (RIFV); (ii) respiratory induced intensity variation (RIIV); (iii) respiratory induced amplitude variation (RIAV). However, this field of research is still little investigated (Wang and den Brinker, 2022). Beyond chest wall movements and modulation of the cardiac activity, uncommonly breathing waveform could be extracted from the shoulder movements (Liu et al., 2017), from nostril movements (Mehta and Sharma, 2020) and consequent head motion (Chen, 2018).

When digital cameras are used, the experimental setting often consists of placing a stable digital video camera in front of the user. The digital cameras used for capturing videos are mainly commercial cameras like webcams (Liu et al., 2017; Massaroni et al., 2018b), and portable device cameras (Karlen et al., 2015; Brieva et al., 2018). Different distances between camera and the user can be employed. The most common is about 1 m that allows recording breathing-related movements with frontal camera of laptop or tablet in a variety of settings (van Gastel et al., 2016; Massaroni et al., 2018a; Antognoli et al., 2019; Rossol et al., 2020). When a digital camera is used, both the framerate and resolution parameters are most important. According to the sampling theorem, a minimum sampling rate of 4 frames per second (hereinafter *fps*) should be guaranteed under the hypothesis that the human breathing frequency lies between 0.1 and 2 Hz (in the case of newborns; Antognoli et al., 2019; Nicolò et al., 2020). Thirty *fps* is the most used setting even because most of cameras work on this range. Considering the video resolutions, typically videos utilize the standard 640 × 480 pixels/frame resolution even if someone employs higher resolutions, such as 1280 × 720 pixels/frame or 1920 × 1080 pixels/frame (Ganfure, 2019; Schrumpf et al., 2019; Massaroni et al., 2019a).

**Figure 3:**
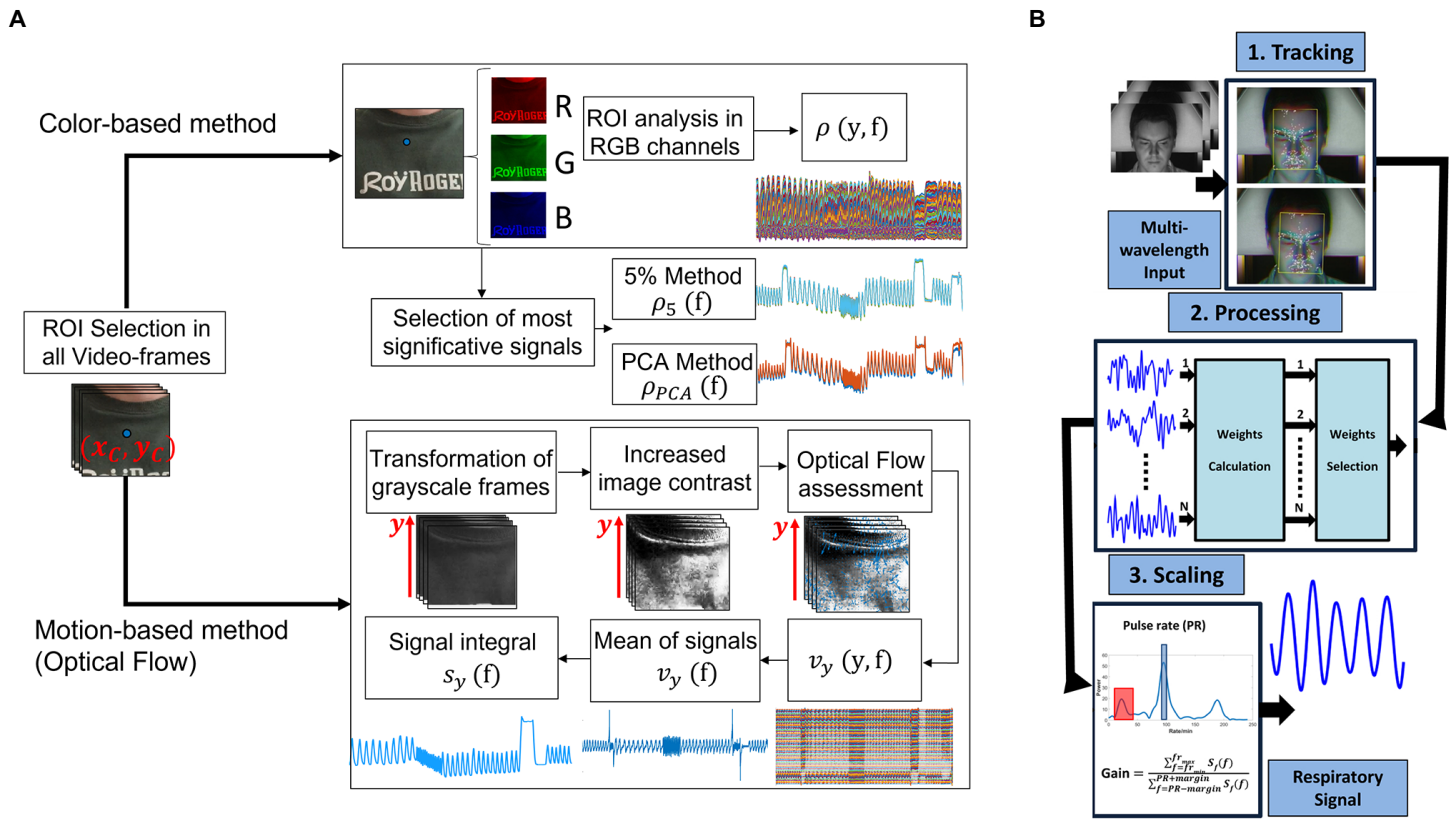
**(A)** Exemplary flowchart with the main steps to extract the breathing pattern from images by analyzing chest wall movements through color-based or motion-based methods (adapted from Romano et al., 2021). **(B)** Framework for respiration detection from rPPG signal (adapted from van Gastel et al., 2016).

The region of interest (ROI) that can be used to extract the respiratory rate and other relevant respiratory features are the torso and the face (Figure 2). In the case of the torso, different areas are used as ROI as the pit of the neck (Massaroni et al., 2018a), the sternum (Massaroni et al., 2019c), the shoulders (Liu et al., 2017). Typically, the individuation of the ROI only in the first frame is sufficient to perform continuous monitoring of the torso movement caused by the breathing activity (Massaroni et al., 2019a) even if approaches based on the continuous tracking of the selected ROI are available in the literature (Shao et al., 2014). Once a ROI has been identified, two different approaches can be used to retrieve a respiratory trace: (i) the analysis of the intensity changes of pixels in the selected ROI (Massaroni et al., 2019a); (ii) the estimation of optical flow along the image gradients (Janssen et al., 2015). The analysis of the intensity changes of the pixels is related to the decomposition of the ROI into three images in the three-color channel (i.e., red, green, and blue). Considering the torso as ROI, the respiratory trace is obtained by averaging the intensity components along with each raw of the ROI (Massaroni et al., 2018b; Figure 3A), while considering the face, the respiratory pattern is retrieved from the rPPG signal since the cardiac activity is modulated by the breathing activity (van Gastel et al., 2016; Chen et al., 2019b; Figure 3B). The estimation of optical flow along the image gradients allows detecting the chest’s motion assuming that the brightness of a feature point in two consecutive frames is the same. The breathing pattern is determined by considering only the flow in the vertical direction (Bartula et al., 2013; Liu et al., 2017; Massaroni et al., 2019c). In some studies, the magnification of the local chest wall movements can be carried out to amplify these movements allowing the detection of little breathing motions (Alinovi et al., 2018; Antognoli et al., 2019). Eulerian Video Magnification (EVM) is the most used algorithm to obtain an amplified video (Wu et al., 2012) which is based on the combination of spatial and temporal processing to accentuate subtle temporal changes in a video. After the application of EVM, the respiratory trace can be obtained by analyzing the intensity changes of pixels (Alinovi et al., 2018; Antognoli et al., 2019) or by analyzing the motion signals retrieved from the magnified videos (see (Alinovi et al., 2018) for more details). When the respiratory pattern is extracted from the head motion, deep-learning techniques (i.e., DeepPhys) can be used to overcome the brightness constancy constraint necessary for optical flow accurate measurements. In (Chen, 2018) the appearance information in video such as the color and texture of the human skin guide the choice on where and how the physiological motions must be estimated. Although there is a reduction in the mean absolute error on average *f_R_* compared with (Tarassenko et al., 2014), this method requires a learning phase.

With digital cameras, the most frequent estimated parameter is *f_R_* (usually measured in breaths per minute—breaths/min) with analysis in the frequency domain to determine the average *f_R_* values or in the time domain for a more in-depth breath-by-breath analysis. Normal values of *f_R_* in adults’ range between 12 breaths/min and 18 breaths/min, while for newborns and infants, it ranges between 20 breaths/min and 30 breaths/min. Abnormal values of *f_R_*, such as tachypnea (*f_R_* is greater than 20 breaths/min) and bradypnea (*f_R_* is less than eight breaths/min) could indicate respiratory problems (Loughlin et al., 2018; Ganfure, 2019). Moreover, in clinical scenarios, an increased value of *f_R_* is a specific predictor of serious events such as cardiac arrest and unplanned intensive care unit admission (Cretikos et al., 2008).

The adequate methods applied on video images acquired with digital cameras can be used to register respiratory activity in a range of applications. A general overview of these applications is provided, focusing on the settings used to monitor respiratory activity.

Most of the studies investigate the potentiality of camera-based technologies for monitoring respiratory activity and estimate *f_R_* in indoor environments to simulate different scenarios in which these techniques could be further applied (Sun, 2011; Bartula et al., 2013; Brieva et al., 2018; Massaroni et al., 2018b, 2019a). The typical experimental setup consists of a camera-based system (e.g., built-in RGB webcam integrated into a laptop, custom-made camera, and commercial camera) and a reference system (e.g., inductive belt, pulse oximeter, and differential digital pressure sensor) to assess the performances of the video-based system. Commonly, the indoor experiments are carried out in structured environment (i.e., laboratory room) and require the subject to be in a rest position, seated in front of the camera, remaining as motionless as possible (Bartula et al., 2013; Ganfure, 2019; Massaroni et al., 2019c). To assess the performance of the techniques, different breathing patterns and thus different values of *f_R_* are simulated, such as: (i) normal breathing or eupnea (*f_R_* between 12 breaths/min and 20 breaths/min); (ii) slow breathing (*f_R_* ~ 12 breaths/min); (ii) tachypnea (*f_R_* > 35 breaths/min; Chatterjee et al., 2016; van Gastel et al., 2016; Massaroni et al., 2019a,c).

Table 1 reports some of the studies dealing with camera-based approaches for estimating *f_R_*. In particular, the following information are reported: (i) sensor, resolution, and framerate; (ii) reference system; (iii) method used to extract the signal from the selected ROI; (iv) field of application, specifying the scenario and population; (v) main obtained results. This table and those that will come later (per each vital sign) evidence a heterogeneity of experimental protocols and conditions as well as different approaches for the estimation of vital signs values on both average or value-by-value basis, and different metrics used to evaluate the performances of methods and measurement systems. This makes some studies not easily comparable.

**Table 1:** Studies using digital cameras for estimating *f_R_* and other respiratory parameters in different fields of application.

Author, Year, Reference	Sensor, resolution, framerate	Reference system	Methods to extract the signal	Fields of application, population, scenario	Main results
Alinovi et al., 2018	RGB camera, 800 × 600 pixel, 30 *fps*	Accelerometer (on adults) and pneumogram (newborns)	Motion-based methods	Test in laboratory and in NICU. 4 adults seated (duration 20′11″), 2 newborns in NICU (duration 8′16″)	Normalized RMSE up to −35 dB.
Antognoli et al., 2019	RGB camera Microsoft LifeCam Cinema, 1280 × 720 pixels, 30 *fps*	Clinical Multiparameter Monitor	Motion-based methods	Tests in NICU. 40 newborns (each recorded up to 1 min)	RMSE = 9.9 breaths/min on average *f_R_.* Bland–Altman MOD ± LOAs = 2.5 ± 11.5 breaths/min
Bartula et al., 2013	Monochrome camera, up to 768 × 576 pixels, 20 *fps*	Thoracic inductance plethysmography	Cross-correlation between consecutive 2D images	Test in laboratory. Five healthy subjects lying down in a bed	*r* = 0.98 Bland–Altman MOD ± LOAs = 0.19 ± 2.46 breaths/min
Brieva et al., 2018	CANON EOS 1300D, 480 × 640 pixels, 30 *fps*	Visual inspection	Motion-based methods	Tests in laboratory. Three subjects in four positions (seat, lying face down, lying face up and lying in fetal position) each recorded up to 1 min	Average MAE% = 2.15
Chatterjee et al., 2016	Consumer-grade camera	Impedance pneumograph	Optical flow	Test in laboratory, 31 healthy subjects, 6 types of breathing experiments (including normal, deep and slow, fast, varying depth of breathing, varying frequency of breathing and breath-holding) different durations (12–155 s)	Bland–Altman MOD ± LOAs = 0.01 ± 5.00 breaths/min on average *f_R_* values
Chen et al., 2019a	RGB camera, 640 × 480 pixels, 10 *fps*	Sleep Respiratory Monitor	Motion tracking of a marker on the chest	Test in laboratory on 20 subjects (seated with a marker on the thoracic surface for 1 min).	*R*^2^ = 0.74
Chen et al., 2019b	Canon camera	Respiratory belt	Intensity of reflected light	Test in laboratory, 6 seated subjects, 30 s	Bland–Altman MOD ± LOAs = 0.04 ± 4.25 bpm
Chen, 2018	RGB camera, 658 × 492, 120 *fps*	Biopotential acquisition unit	Motion-based methods	Tests in laboratory on 25 subjects.	MAE < 3.1 breaths/min for participant dependent performance; MAE < 5 breaths/min for participant independent performance. The results are obtained by using CNN approach.
Ganfure, 2019	Laptop camera, 640 × 480 pixels, 22 *fps*	breathing rate counted manually	Optical flow	Test in laboratory on 5 subjects. Distance ranging between 0.5 m and 2 m	Average percentage error of 15.44%
Hassan et al., 2017b	RGB camera, 780 × 580 pixels, 61 *fps*	Respiratory belt	Breathing waveform from rPPG signal	Tests on videos from database (MAHNOB-HCI) on 27 subjects. Distance of about 0.4 m	Bland–Altman MOD ± LOAs = 0.58 ± 4.92 breaths/min
Janssen et al., 2015	RGB camera, 768 × 576 pixels	Impedance pneumography for adults and ECG for newborns	Optical flow	Test in laboratory on 4 subjects. Tests in NICU on 2 newborns	Bland–Altman MOD ± LOAs = 0.07 ± 2.74 breaths/min
Jorge et al., 2017	A camera with 3 CCD optical sensor, 1628 × 1236 pixels, 20.3 *fps*	Clinical Multiparameter Monitor	Intensity of light	Test in NICU on 30 preterm infants.	N.A.
Karlen et al., 2015	Mobile phone camera (Camera Oximeter), 240 × 320 pixels, 20 *fps*	Capnography	Breathing waveform from rPPG signal	Test in laboratory on 19 subjects.	RMSE = 6 breaths/min
Liu et al., 2017	Logitech C905 webcam, 960 × 720 pixels, 30 *fps*	Spirometer	Optical flow	Test in laboratory on 16 subjects.	RMSE ≤0.27 l
Massaroni et al., 2018b	RGB laptop webcam, 1280 × 720 pixels, 30 *fps*	Differential digital pressure sensor	Intensity of reflected light	Test in laboratory on 6 subjects. Distance of about 1.2 m	Bland–Altman MOD ± LOAs = 0.02 ± 2.37 breaths/min
Massaroni et al., 2019a	RGB laptop webcam, 1280 × 720 pixels, 30 *fps*	Head-mounted wearable device	Intensity of reflected light	Test in laboratory on 12 subjects, seated in front of the camera at about 1.2 m	Bland–Altman MOD ± LOAs = −0.01 ± 1.02 breaths/min
Massaroni et al., 2019c	RGB laptop webcam, 1280 × 720 pixels, 30 *fps*	Head-mounted wearable device	Intensity of reflected light, optical flow	Test in laboratory on 8 subjects, seated in front of the camera at about 1.2 m	MAE < 1 breaths/min for optical flow, MAE < 3 breaths/min for intensity of light
Mateu-Mateus et al., 2021	Logitech C920 camera, 1080 × 1920 pixels, 15 *fps*	Respiratory belt	Motion tracking	Tests in laboratory on 21 subjects, in front of the camera at about 0.7 m	MAPE <4% for constrained respiration, MAPE <6% for unconstrained respiration
Mehta and Sharma, 2020	RGB camera, 720 × 586 pixels, 50 *fps*	Respiratory belt	Breathing waveform from rPPG signal	Tests on 13 subjects	RMSE = 4.01 breaths/min for window length of 30 s, RMSE = 3.63 breaths/min for window length of 60 s
Reyes et al., 2017	Smartphone, 1080 pixels, 30 fps	Spirometer	Intensity of reflected light	Tests in laboratory on 15 subjects in a stand position, performing different volume ranging from 300 ml to 3 l. Distance of about 0.6 m	RMSE = 0.182 l
Rossol et al., 2020	IP camera, 320 × 180 pixels, 10 *fps*	ECG contact-based	Motion-based methods	Tests in NICU on 2 newborns	Bland–Altman MOD ± LOAs = −1.3 ± 12.2 breaths/min
Schrumpf et al., 2019	RGB camera, 1920 × 1080 pixels, 120 *fps*	Movements of the chest from video	Breathing waveform from rPPG signal	Test in laboratory on subjects in supine position	Error in the range of ±0.50 breaths/min on the average *f_R_* values
Shao et al., 2014	RGB camera (Pike color camera), 640 × 480 pixels, 208 *fps*	Respiratory belt and Oxycon metabolic analysis instrument	Motion tracking of the shoulder	Tests in laboratory on 10 subjects, simulating different breathing patterns	Bland–Altman MOD ± LOAs =0.02 ± 2.45 breaths/min
Sun, 2011	Monochrome camera, up to 1280 × 1024 pixels, 20 *fps*	N.A.	Breathing waveform from rPPG signal	Test in laboratory on 1 subject during activity routine for about 12 min. Distance of about 0.4 m	Results show that there is an increase of the *f_R_* values during the performed exercises.
Tarassenko et al., 2014	RGB camera, 5 Mpixels, 12 *fps*	Chest belt	Intensity of reflected light	Tests in a dialysis room. 46 subjects	Values estimated with the camera-based system are comparable with that obtained from the reference system
van Gastel et al., 2016	RGB camera, 768 × 576 pixels, 20 *fps*	Pulse oximeter	Breathing waveform from rPPG signal	Test in laboratory on 3 adults (duration 120–150 s, simulating different breathings). Tests in NICU on 2 newborns (spontaneous breathing). Camera distance about 1 m	RMSE = 2.67 breaths/min, MAE = 1.74 breaths/min for guided breathing. RMSE = 9.35 breaths/min, MAE = 4.72 breaths/min for spontaneous breathing

The sensor, resolution, and framerate of the proposed systems are specified as well as the methods used to extract the respiratory waveform. Details on the fields of application and enrolled populations, as well as details on the reference system are provided. The main results of each study are reported in the last column (RMSE, Root mean square error; MOD, Mean of difference; LOAs, Limit of agreements; *r*, Correlation coefficient; MAE, Mean absolute error; MAPE, Mean percentage absolute error; *R*^2^, Determination coefficient; N.A, means not available; CNN, Convolutional neural network).

Looking at the clinical settings applications, several of the available studies explore the potentiality of these camera-based techniques to detect the breathing activity and to monitor *f_R_* in the Neonatal Intensive Care Unit (i.e., NICU) on newborns and/or preterm infants (Janssen et al., 2015; Alinovi et al., 2018; Antognoli et al., 2019; Rossol et al., 2020). Mainly, the system involves one camera to record the video and a reference instrument to carry out a validation study (e.g., multiparameter monitor, standard hospital ECG impedance pneumography, or elastic belt for pneumogram recording and in general strain sensors). The camera must be adequately positioned to capture the chest of the baby at a distance ranging between 50 cm to about 2 m (Alinovi et al., 2018; Antognoli et al., 2019; Rossol et al., 2020). Due to the small-amplitude chest wall movements of the babies, different approaches dealing with video magnification can be used to extract respiratory waveforms. Rossol et al. (2020) have used a micromotion and stationary detection algorithm to amplify and track movements by averaging background noise; authors found a root mean square error (RMSE) of 6.36 breaths/min comparing the *f_R_* values extracted from the signal’s video and the values recorded by the electronic medical record. In (Antognoli et al., 2019) the *f_R_* value has been extracted as the highest Power Spectrum Density (PSD) peaks in the typical *f_R_* interval, and an RMSE of 4.9 breaths/min is reported. A wider range of volunteers was investigated in Alinovi et al. who tested the system both in adults and in newborns with a motion magnification algorithm. Results show that the proposed method can be promising in estimating *f_R_* when compared to the values obtained from the reference systems (i.e., accelerometer for adults, pneumography for newborns; Alinovi et al., 2018). The application of a camera system for respiratory monitoring in a dialysis room has been tested in (Tarassenko et al., 2014), where authors recorded a 4 h video for each volunteer. The system allows the estimation of *f_R_* by considering the face as ROI and extracting the respiratory trace from the rPPG signal. Results show that the values of *f_R_* estimated from the breathing-synchronous changes in the amplitude of the reflected PPG signal are comparable with that derived from the reference system (i.e., chest belt). Mainly, in the applications described above, the breathing pattern and the respiratory rate are extracted from the chest wall movements of the subject by using different methods for video analysis, that rely with the video magnification such as EVM, motion magnification algorithm, etc. (Alinovi et al., 2018; Antognoli et al., 2019; Rossol et al., 2020) prior to the extraction of the respiratory pattern. Since the tests have been carried out in clinical settings on newborns in NICU or patients in dialysis, the *f_R_* extracted are that typical of the normal breathing (e.g., for newborns *f_R_* ranges between 30 breaths/min and 70 breaths/min). Camera-based methods find interesting applications even outside the clinical scenarios. Of particular interest is the study of Mateu-Mateus et al. (2021) who proposed a video-based method to record respiratory activity by detecting the movement of three small patterns attached on a seatbelt fastened by the subject for detecting drowsiness while driving in a simulated cabin car. Comparing the proposed method with the reference system (i.e., respiratory chest belt), a mean percentage absolute error (MAPE) lower than 4% for constrained respiration and a MAPE<6% for unconstrained respiration are reported. Interestingly authors in (Sun, 2011) carried out tests on healthy volunteers to investigate respiratory variations during the simulation of various exercise levels (i.e., cycling exercise at moderate and high-intensity levels) and recovery from the exercise. In this case, the breathing pattern has been extracted from the color intensity changes of the facial skin (based on color intensity methods). Results show that the values of *f_R_* increase from 18 breaths/min to 24 breaths/min during the moderate intensity level and assumes the highest value (up to 30 breaths/min) in the high-intensity level, even if these results were not compared against reference values. In (Zhang et al., 2014), the authors investigated the performance of a camera-based system to compute *f_R_* from drivers’ face video with the aim to estimate the mental status of the subject with a real-time measurement system framework running on a laptop. As reported in (Grassmann et al., 2016), respiratory activity and thus respiratory rate are influenced by cognitive load and stress. Similarly, McDuff et al. estimate the cognitive stress of different users seated in front of a camera while doing some computer tasks by measuring physiological parameters (among others, the *f_R_* and other cardiovascular parameters). A digital camera was used to detect the rPPG waveform from the face and then to estimate the *f_R_*. This enabling technology allows finding no significant changes in the values of *f_R_*, but overall changes in all the other cardiovascular parameters extracted from the same videos (McDuff et al., 2016).

Besides *f_R_*, other respiratory-related parameters can be retrieved from the breathing pattern extracted from a video. Interesting is the study presented in (Reyes et al., 2017) where the air volumes exchange like tidal volume (*V*_T_) was estimated by digital videos, as well as the exhalation flow rate (Shao et al., 2014) or forced expiratory volume in the first second (FEV1) and forced vital capacity (FVC; Liu et al., 2017). *V*_T_ is defined as the air moved with each breath and provides information about the depth of the breathing (Reyes et al., 2017), while the exhalation flow rate is proportional to the subject’s metabolic rate and can be obtained by dividing the exhalation breath volume by exhalation time (Shao et al., 2014). FEV1 and FVC are important parameters for the diagnosis and management of asthma and chronic obtrusive pulmonary disease (COPD) that can be derived from the flow volume curve (Liu et al., 2017). To obtain these respiratory-related parameters, a calibration procedure is needed (Shao et al., 2014; Reyes et al., 2017), and according to the monitored parameter, a different reference instrument (e.g., spirometer and metabolic analysis instrument) is used to assess the performances of the camera-based systems. Regarding the calibration procedure, authors in (Reyes et al., 2017) performed a linear regression between the reference *V*_T_ registered by the spirometer and the absolute peak-to-peak amplitude of chest movement signal.

### Heart Rate and Other Cardiac Parameters

The measurement of cardiac parameters with digital camera is mainly (but not exclusively) based on the registration of the rPPG signal from light-exposed skin regions. Typically, the face is the preferred anatomical site since the cyclical movement of the blood from the heart to the head *via* the abdominal aorta and the carotid arteries causes skin color changes of the face and subtle head oscillations. The main suitable sites for the estimation of these parameters are the cheeks, which include a higher proportion of capillaries, and the forehead since it is not affected by muscle movements (Hassan et al., 2017a). The optical properties of the facial skin are determined by the presence of different chromophores in the layers of the skin, due to the exchanges of gases through the cardiorespiratory action. Mainly, the skin color is given by the presence of melanin in the epidermis and the hematic pigments (i.e., hemoglobin, oxyhemoglobin, beta carotene, and bilirubin) present in the dermis/hypodermis vascular plexus (Davila et al., 2017). The dominating chromophore in the blood is hemoglobin (percentage of around 45%). The spectrum of light absorbed by the hemoglobin in the blood is different for the oxygenated and deoxygenated states: blood absorbs light more than the surrounding tissues since it is dark and opaque. Furthermore, the oxygenated blood is lighter in color than the deoxygenated blood (Al-Naji et al., 2017). Moreover, considering that the inflow of the blood from the heart to the head causes subtle head movements at each pulse, the cardiac information can be retrieved by detecting the head motions (Balakrishnan et al., 2013; Shan and Yu, 2013).

When a video-based method must be used to monitor HR, a fixed digital camera must be positioned in front of the user. As in the case of *f_R_* estimation, different cameras can be used (Poh et al., 2011; Mestha et al., 2014; Han et al., 2015; Zhang et al., 2017; Maji et al., 2020; Yang et al., 2021a) as well as different user-camera distances (Scalise et al., 2012; Sanyal and Nundy, 2018; Song et al., 2020). The most common distance is about 1 m that allows recording the facial region with the frontal camera of laptop or tablet in several settings (Rasche et al., 2016; Hassan et al., 2017b; Shoushan et al., 2021). Taking into account that the possible HR values ranges from 40 [bradycardia in young users (Kawachi et al., 1995; Longin et al., 2005; Sharma et al., 2015)] to around 220 bpm (which is the maximum HR value in young users or in newborns), a minimum sampling rate of 8 *fps* should be guaranteed (sampling theorem). However, considering the analysis of heart rate variability (HRV), different studies investigated the minimum sampling frequency (*f_s_*) that should be assured (Choi and Shin, 2017; Liu et al., 2020; Béres and Hejjel, 2021). Authors in (Choi and Shin, 2017) showed that the minimum *f_s_* depends on the HRV variables which can be obtained (e.g., *f_s_* > 10 Hz for LF/HF, *f_s_* > 20 Hz for NN50 and pNN50; see Choi and Shin (2017) for further details). Another study assessed the minimum *f_s_* by analyzing HRV signals obtained from PPG signal which may or may not be interpolated. Results showed that SDNN (Standard Deviation of PP interval) and RMSSD (Root Mean Square of Successive PP differences) require an *f_s_* of 50 Hz without interpolation, which can be reduced by interpolation at 10 Hz and 20 Hz, respectively (Béres and Hejjel, 2021).

Despite everything, 30 *fps* is the most used framerate, whereas a video resolution of 640 × 480 pixels/frame resolution is the most common, even if higher resolutions (e.g., 1280 × 720 pixels/frame or 1920 × 1080 pixels/frame) are employed in some studies dealing with HR estimation from video images (Shan and Yu, 2013; Kuo et al., 2015; Jain et al., 2016; Hassan et al., 2017b; Antognoli et al., 2019).

**Figure 4:**
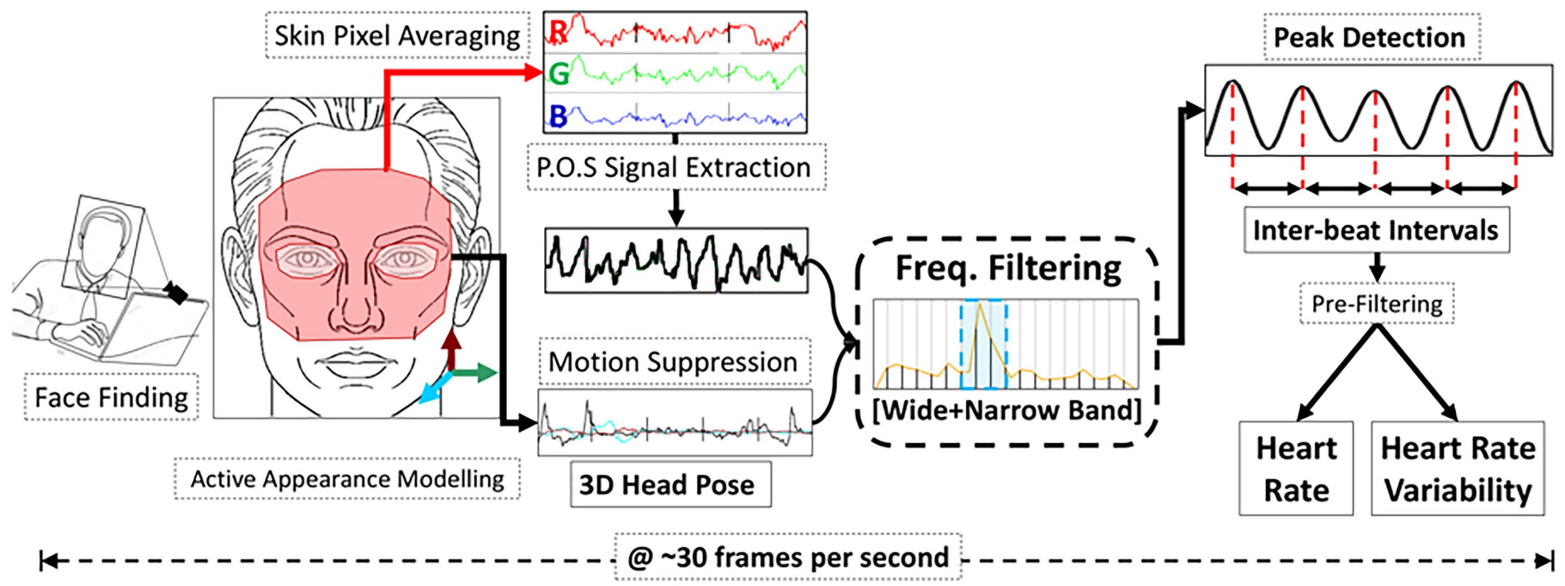
An example of the pipeline used to extract HR and heart rate variability from rPPG signal extracted through a color-based method based on POS post-processing algorithm (from Gudi et al., 2020).

The ROI used to extract the HR and other related cardiac parameters is typical the facial region. In more detail, different areas can be used as ROI, like forehead, left cheek, right cheek, or the whole face (Favilla et al., 2019; Lamba and Virmani, 2020; Shoushan et al., 2021). Moreover, other studies investigated alternative regions of the skin exposed to light like the palm hand (Cennini et al., 2010; Sun et al., 2012; Sereevoravitgul and Kondo, 2014; Lu et al., 2018; van der Kooij and Naber, 2019). Typically, the ROI identified in the first frame of the video is considered the same in all the video’s frames, even if different studies employed approaches based on ROI tracking (Tasli et al., 2014) and on the use of multiple cameras to frame different positions of the face (Mcduff et al., 2017). Once a ROI has been identified, different approaches can be used to retrieve the rPPG signal valuable for the estimation of HR. There are hundreds of post-processing algorithms in this field. A detailed description of each is beyond the scope of this review; the authors recommend the following review papers on this topic (Hassan et al., 2017a; Rouast et al., 2018; Wang et al., 2018). Among the available post-processing algorithms, blind source separation (BSS) based methods and skin optical reflection model-based methods are the two main categories (see an example in Figure 4). Even combination of color channels and combination of spatially separated regions are feasible approaches (Wedekind et al., 2017). The BSS-based methods assume the source signal to satisfy some statistical nature, such as independence or correlation. The most used algorithms are the Independent Component Analysis (ICA) and the Principal Component Analysis (PCA; Poh et al., 2011; Lewandowska and Nowak, 2012; Zhang et al., 2014). ICA assumes that the set of observations (i.e., signals captured from RGB sensors) is composed of linear mixtures of the source signals (i.e., reflected plethysmographic signal; Poh et al., 2010, 2011). PCA allows recovering the directions along which the data have maximum variance, and the goal is to represent this data as a set of new orthogonal variables named principal components (Abdi and Williams, 2010). Different from the BSS-based methods, the skin optical reflection methods assume the pulse signal to satisfy a skin optical reflection mode. The most used method is based on the analysis of the intensity changes of the pixels of the selected ROI in the green channel since it is related to the high absorption of the light by the hemoglobin in the range of the visible light (Tasli et al., 2014; Kuo et al., 2015; Rasche et al., 2016; Maji et al., 2020). However, the author in (de Haan and Jeanne, 2013) firstly proposed a chrominance-based signal processing method (CHROM) to clearly extract pulse signal against specular and motion artifacts by projecting RGB channels into a chrominance subspace where the motion component is eliminated. Another method that can be used to retrieve the pulse signal is the POS (i.e., plane orthogonal to the skin) method which uses a different projection orthogonal to the skin tone compared to CHROM method, and it is considered to be more robust in complex illumination scenarios (Wang et al., 2017). In few studies, the analysis of the video is carried out in a different color space by transforming the video RGB into a HSV (Hue, Saturation, and Value) color space (Sanyal and Nundy, 2018) or in YCbCr space (Zhang et al., 2017). Furthermore, in the last years, there is a growing interest in the use of machine learning and deep-learning techniques for the estimation of HR. Authors in (Ni et al., 2021) proposed an overview of the main deep-learning methods that can be used to obtain an estimate of HR, trying to provide guidelines regarding the possible step in rPPG signal analysis where deep-learning can be used (e.g., from the extraction of the signal to the estimation of HR). As an example, authors in (Chaichulee et al., 2019) used Deep Learning to identify the patient in the field of the view of the camera and which pixels correspond to the skin. In another study, a comparison between the use of different types of deep learning techniques (i.e., Deepphys, rPPGNet, Physnet) and the traditional methods (i.e., CHROM, POS, ICA, and Green Channel) was performed in the estimation of HR (Yang et al., 2021b) under varying lighting conditions. Deep learning-based methods demonstrated superior performance compared to traditional methods (in terms of mean absolute error—MAE—and RMSE of average HR), but the latter are generally more robust to the light variations (Yang et al., 2021b).

With digital cameras, the most frequent estimated parameter is HR (measured in beats per minute—bpm or, less commonly, pulse per minute—ppm). Normal values of HR range between 60 bpm and 100 bpm in adults and range between 70 bpm and 190 bpm in children (Kebe et al., 2020). Abnormal values of HR are related to values greater than 100 bpm (i.e., tachycardia) and lower than 60 bpm (i.e., bradycardia). These abnormalities occur when the normal electrical impulse that controls the pumping action of the heart is interrupted by physiological and pathological factors (Hassan et al., 2017a).

The methods applied on video images acquired with digital cameras can be used to detect cardiac activity in a range of applications. In this section, a general overview of these applications is provided, focusing on the settings used to monitor cardiac activity.

Most studies investigate the capabilities of camera-based technologies for monitoring cardiac activity and estimating HR in indoor environments to simulate different scenarios in which these technologies could be applied (Balakrishnan et al., 2013; Tasli et al., 2014; Lamba and Virmani, 2020; Maji et al., 2020; Song et al., 2020; Shoushan et al., 2021). In few studies, experiments to simulate training sessions (e.g., running and cycling) have been performed (Sun, 2011; Tasli et al., 2014), and other studies investigate the potentiality of applying non-contact technologies in the automotive field (Zhang et al., 2014; Huan Qi, 2015; Kuo et al., 2015). The typical experimental setup is composed of one camera and a reference system (e.g., chest belt, pulse oximeter, and standard ECG) to assess the performances of the video-based system. The camera is placed at a distance ranging from 0.5 m to 3 m. Commonly, experiments are carried out in structured environments (e.g., a lab) and require the subject to be in a rest position, typically seated in front of the camera, remaining as motionless as possible (Sanyal and Nundy, 2018; Favilla et al., 2019; Maji et al., 2020). In a limited number of studies, experiments were carried out in upright position (Sanyal and Nundy, 2018). Table 2 reports some of the studies dealing with camera-based approaches for estimating HR.

**Table 2:** Studies using digital cameras for estimating HR and other cardiac-related parameters in different fields of application.

Author, Year, Reference	Sensor, resolution, framerate	Reference system	Methods to extract the signal	Fields of application, population, scenario	Main results
Aarts et al., 2013	RGB camera, 300 × 300 pixels, up to 30 *fps*	ECG	GC	Tests in NICU on 19 infants. Distance of about 1 m	Bland–Altman MOD ± LOAs = 0.3 ± 5.3 bpm
Al-naji and Chahl, 2017	Nikon D5300 DSLR camera, 1080 × 720 pixels, 30 *fps*	Pulse oximeter	Motion-based methods	Test in laboratory on 10 subjects. Distance of about 0.5 m	Bland–Altman for DVM, MOD ± LOAs = 0.47 ± 1.8 bpm, Bland–Altman for EVM, MOD ± LOAs = 0.69 ± 2.4 bpm
Antognoli et al., 2019	RGB camera, 1280 × 720 pixels, 30 *fps*	Clinical Multiparameter Monitor	Motion-based methods - Intensity of reflected light	Tests in NICU on 40 newborns (each recorded up to 1 min). Distance of about 0.5 m	RMSE = 6.8 bpm
Balakrishnan et al., 2013	Camera Panasonic Lumix GF2, 1280 × 720 pixels, 30 *fps*	ECG	Motion-based (feature tracking)	Test in laboratory on 18 subjects. Tests on 1 newborn in NICU	N.A.
Favilla et al., 2019	Monochrome camera, 352 × 224 pixels, 133 *fps*	ECG	ICA	Tests in laboratory on 30 subjects, considering 3 ROIs, downsampling the framerate and the acquisition time	Results show that the forehead has lower errors that the cheek; the downsampling and the video duration do not affect HR values
Guo et al., 2014	Laptop camera, 15 *fps*		IVA	Tests in laboratory to simulate driving task.	Average Error = 0.7 bpm
Han et al., 2015	Laptop camera and smartphone camera, 640 × 480 pixels, 30 *fps*	Pulse oximeter	ICA	Tests in laboratory on 10 healthy subjects	Bland–Altman Laptop vs. reference: MOD ± LOAs = 0.40 ± 3.72 bpm Laptop vs. smartphone: MOD ± LOAs = 0.01 ± 2.92 bpm
Hassan et al., 2017b	Logitech C920 HD pro webcam, 1080 × 1920 pixels, 30 *fps*	Pulse oximeter	GC	Test in laboratory on 45 subjects simulating three scenarios (neutral, motion variance, skin tone variance). Distance of about 0.8 m	RMSE = 4.97 bpm (neutral scenario); RMSE = 7.28 bpm (motion variance)
Jain et al., 2016	Sony HDR-CX405 camcorder, 1920 × 1080 pixels, 50 *fps*	Digital Blood pressure monitor	PCA	Tests in laboratory on 45 subjects for 1 min, seated with eyes closed.	MAE =1.73 bpm
Krolak, 2017	Laptop camera, 640 × 480 pixels, 25 *fps*	ECG	PCA	Tests in laboratory on 12 subjects with different skin tones for 1 min.	Results show that the different skin tone affects the performance of the vision-based system.
Kuo et al., 2015	Two video cameras, 1920 × 1080 pixels, 29 *fps*	Chest belt	GC	Tests during a driving task on 10 subjects.	Mean error ranging from −25.77 bpm to 35.34 bpm with reference HR ranging from 66 to 100 bpm.
Kwon et al., 2012	Smartphone camera, 640 × 480 pixels, 30 *fps*	ECG	ICA	Tests in laboratory on 10 subjects. Distance of about 0.3 m	Average error = 1.47%
Lamba and Virmani, 2020	RGB camera, 640 × 480 pixels, 20 Hz	Reference value computed using Biosppy package	GC	Test on videos from the dataset COHFACE. 15 subjects	RMSE =8 0.35 bpm from HR extracted from cheeks, RMSE = 12.21 bpm from cheeks and noise
Maji et al., 2020	RGB laptop camera, 1280 × 720 pixels, 30 Hz	Pulse oximeter	Intensity changes, PCA	Test in laboratory on 6 subjects. Distance of about 1.2 m	Absolute error < 3 bpm (from carotid region).
Mestha et al., 2014	Webcam, 640 × 480 pixels	Clinical Multiparameter monitor	GC	Tests in NICU on 8 newborns. Distance of ~1 m	Bland–Altman MOD ± LOAs = 2.52 ± 5.48 bpm
Poh et al., 2010	Laptop camera, 640 × 480 pixels, 15 *fps*	BVP sensor	GC, ICA	Tests in laboratory on 12 subjects at rest and during motion. Distance of about 0.5 m	RMSE = 6.00 bpm (GC at rest); RMSE = 2.29 bpm (ICA at rest). RMSE = 19.36 bpm (GC - motion); RMSE = 4.63 bpm (ICA - motion)
Poh et al., 2011	Laptop camera, 640 × 480 pixels, 15 *fps*	BVP sensor	ICA	Test in laboratory on 12 subjects. Distance of about 0.5 m	RMSE = 1.24 bpm
Huan Qi, 2015	Webcam	Pulse oximeter	Joint BSS	Test in laboratory (distance of 1.5 m) and during driving on 16 subjects	Results show that the proposed framework is promising for HR estimation
Rasche et al., 2016	Two cameras, 420 × 320 pixels, 100 *fps*	Multiparameter monitor	GC	Tests in cardiac surgical ICU. Distance between 0.6 m and 1 m	83% of the results shows a mean error of −0.1 ± 0.8 bpm
Sanyal and Nundy, 2018	Smartphone camera	Pulse oximeter	GC and analysis of the Hue values in HSV color space	Test in laboratory on 25 subjects with different skin tones. Distance of ~0.5 m	RMSE = 0.89 bpm (HUE), RMSE = 0.91 bpm (GC)
Scalise et al., 2012	RGB camera, 640 × 480 pixels, 30 *fps*	ECG	ICA	Tests in NICU on 7 patients. Distance of 0.2 m and a light source placed at about 1 m	Bland–Altman MOD ± LOAs = −0.90 ± 8.89 bpm
Shan and Yu, 2013	Smartphone camera, 1280 × 720 pixels, 30 *fps*	Blood pressure monitor	Feature tracking (BCG)	Tests in laboratory on 9 subjects. Distance of about 0.4 m	Mean error = 0.86%
Shao et al., 2014	3 digital cameras (Logitech Webcam, Pike black and white camera, Pike color camera)	Pulse oximeter	GC	Test in laboratory on 10 subjects. Distance of about 0.5 m	Bland–Altman MOD ± LOAs = 0.86 ± 3.33 bpm
Shoushan et al., 2021	Laptop webcam, 340 × 480 pixels, 30 *fps* Smartphone camera, 1920 × 1080 pixels, 30 *fps*	ECG	POS	Test in laboratory on 9 subjects performing three maneuvers (spontaneous breathing, metronome breathing, forced breathing). Distance of ~1 m	Bland–Altman smartphone MOD ± LOAs = −0.25 ± 1.59 bpm Bland–Altman webcam MOD ± LOAs = 0.05 ± 1.91 bpm
Singh et al., 2017	Laptop webcam, 15 *fps*	Stethoscope	ICA	Tests in laboratory on 3 subjects.	Obtained results with an accuracy between 94 and 95%
Song et al., 2020	GoPro camera, 2704 × 1520 pixels, 30 *fps*	ECG	GC, ICA, CHROM; POS, BCG	Tests in laboratory on 15 subjects. Distances from 0.5 m up to 3.0 m under various resolutions.	Results show that the resolution affects the quality of the rPPG signal at distances less of 1 m.
Sun, 2011	Monochrome CMOS camera, 1280 × 1024 pixels	Pulse oximeter	ICA	Test in laboratory on 12 subjects during cycling exercise at 15 km/h (ex.1) and 25 km/h (ex.2)	Bland–Altman MOD ± LOAs = −0.78 ± 1.51 bpm (ex1) MOD ± LOAs = −0.55 ± 1.87 bpm (ex2)
Sun et al., 2012	Monochrome camera, 256 × 384 pixels, 200 *fps*	Pulse oximeter	Color-based	Tests in laboratory on 10 subjects	Bland–Altman MOD ± LOAs = −0.04 ± 2.97 bpm
Tarassenko et al., 2014	RGB camera, 5 Mpixels, 12 *fps*	Pulse oximeter	GC	Tests in a dialysis room on 46 subjects	Values estimated with the camera-based system are comparable with that obtained from the reference system
Tasli et al., 2014	Camera, 720 × 1280 pixels, 30 *fps*	Pulse oximeter	GC, ICA	Tests in laboratory on 10 subjects at rest (ex.1) and after running exercise (ex.2).	Mean error between 2.9 bpm and 4.3 bpm for ex.1, and between 4.2 bpm and 5.6 bpm for ex.2
Yang et al., 2021b	Logitech webcam, 640 × 480, 20 *fps*	Pulse oximeter	GC, ICA, POS, CHROM	Tests in laboratory on 12 subjects under different lighting conditions. Distance of about 1 m. Test on videos from the dataset UBFC-rPPG	MAE = 2.33 bpm for Physnet using the database UBFC-rPPG. Results show that varying lighting conditions affect the performance of DL methods
Zhang et al., 2017	Webcam, 640 × 480 pixels, 10 *fps*	Pulse oximeter	ICA	Test in laboratory (under different scenarios) on 100 subjects and during driving (1 subject). Distance of about 0.5 m	RMSE = 1.8 bpm

The sensor, resolution, and framerate of the proposed systems are specified as well as the methods used to extract the rPPG waveform. Details on the fields of application and enrolled populations, as well as details on the reference systems, are provided. The main results of each study are reported in the last column (GC, Green channel; ICA, Independent component analysis; IVA, Independent vector analysis; PCA, Principal component analysis; POS, Plane orthogonal to skin; CHROM, chrominance-based signal processing method; DL, Deep learning; RMSE, Root mean square error; MOD, Mean of difference; LOAs, Limit of agreements; MAE, Mean absolute error, N.A, means not available).

As for the case of *f_R_*, considering the clinical applications, most of the available studies tested the video-based techniques to monitor HR in the NICU, on newborns and/or preterm infants on which contact-based systems can cause possible skin irritations and discomfort (Scalise et al., 2012; Antognoli et al., 2019). Mainly, the system is composed of a camera placed at a distance ranging from 20 cm (Scalise et al., 2012) to around 1 m (Mestha et al., 2014; Rasche et al., 2016) from the patient. Authors in (Scalise et al., 2012) have used the ICA algorithm to extract the blood volume pulse signal, and HR has been estimated by computing the Power Spectrum Density (PSD), a frequency domain analysis, of the extracted signal considering the frequency at which occur the maximum peak of the PSD and by multiplying this value for 60. Comparing the obtained results with that obtained from the reference system, a bias of −0.9 bpm has been found. Mestha et al. have estimated HR from the recorded video of neonates by making a ROI tracking of the face. The Green Channel (GC) component of the signal has been analyzed, and a bias of 2.52 bpm has been obtained compared to data resulting from the reference system (i.e., a multiparameter monitor; Mestha et al., 2014). Outside the NICU, camera-based systems have been tested to monitor patients in the cardiac surgical Intensive Care Unit (i.e., ICU) after elective cardiac surgery has been tested in (Rasche et al., 2016). The camera was placed to record the face and the upper body of the patient: the frontal face skin was considered as ROI, and the analysis of the G channel component of the signal was carried out. Comparing the estimated value of HR with the value taken from the ECG reference monitoring, a mean difference of −0.1 ± 0.8 bpm has been obtained. Mainly, in this kind of application, the cardiac signal is derived by using methods based on the variations of the color intensity of the facial skin (i.e., rPPG). The algorithms used to extract the rPPG signal are focused on the G channel component and ICA analysis. To estimate the values of HR, the rPPG signal was filtered in a frequency range typical of normal values of HR (i.e., 42–120 bpm). Even in an occupational scenario, digital cameras can be used to monitor HR, as in the automotive field (Zhang et al., 2014). To test the performance of the system, the authors tested the real-time system during a driving session under normal daylight conditions. The value of HR was estimated from the cardiac signal extracted from the whole face of the subject; comparing the HR values against those from the reference system (i.e., pulse oximeter), an RMSE of 4.15 bpm was found. Differently, Tasli et al. (2014) used a camera-based system to monitor HR by using a ROI tracking algorithm to detect the face of the subject during a simulated training session (i.e., a short session of running) before sitting in front of the camera. Results show that immediately after the exercise, HR value is above 120 bpm, and then it decreases slowly until 50 bpm as the subject’s state of fatigue is alleviated. An average error between 4.2 bpm and 5.6 bpm was obtained comparing the proposed algorithm with the EVM method. The application of a camera-based system during a cycling exercise at two different speeds (i.e., 15 km/h and 25 km/h) was tested in (Sun, 2011). Results showed reasonable outcomes during the moderate cycling exercise at 15 km/h, where HR gradually increases from 87 bpm to about 105 bpm, and its value reaches 120 bpm during the high-intensity exercise at 25 km/h.

Besides heart rate, other cardiac-related parameters can be estimated with enormous potentialities in neuroscience and physiology sciences. Among others, literature evidenced the possibility to estimate HRV, which is mainly related to mental stress, and the inter-beat interval (IBI). These two parameters, along with the HR, contribute to assessing the physical and psychological status of an individual (Davila et al., 2017). HRV represents the fluctuations in the time intervals between adjacent heartbeats, and it is an index of the adaptation of the heart to circumstances; thus, it is an indicator of adaptation in athletes or fatigue in drivers (Favilla et al., 2019). IBI is the time between consecutive heartbeats, expressed in milliseconds, and is calculated as the consecutive difference of time component of the R peaks (Davila et al., 2017). Considering the use of contactless methods based on cameras, HRV and IBI can be obtained from the rPPG signal extracted from a video of the subject (Poh et al., 2010; Davila et al., 2017).

### Blood Oxygen Saturation

The measurement of blood oxygen saturation (SpO_2_) is related to the concentration of hemoglobin (Hb) and oxygenated hemoglobin (HbO_2_) in the blood, as it is calculated as the ratio between HbO_2_ and the total amount of Hb (i.e., deoxygenated and oxygenated hemoglobin; Foo et al., 2013). The main method used to estimate SpO_2_ is the pulse oximeter, whose working principle is associated with the different absorption of light by HbO_2_ and Hb at two different wavelengths (normally *λ*_1_ = 660 nm that corresponds to the red light, and *λ*_2_ = 940 nm corresponding to the infrared (IR) light; Foo et al., 2013). Values between 95 and 100% are considered normal (O’Brien et al., 2000). A lower percentage of oxygen (<95%) indicates hypoxia and causes insufficient oxygen supply to the human body (Dias and Cunha, 2018). A problem in the measurement of SpO_2_ is when the patient is anemic: even if the anemic patient has a lower level of hemoglobin, the value of SpO_2_ may be in the normal range (Elliott and Coventry, 2012).

The typical experimental setup consists of the same items as the one used for estimating HR from videos (see section “Heart Rate and Other Cardiac Parameters”). Distances ranging between 0.5 m and 1.5 m from the user are employed, even if the most common is about 1 m (Kong et al., 2013; Guazzi et al., 2015; de Fatima Galvao Rosa and Betini, 2020), allowing the recording of the cardiac activity to retrieve the rPPG signal valuable for the estimation of the SpO_2_. Thirty *fps* is the most commonly used framerate since it is the typical value of the majority of the commercial camera, even if in some studies lower framerates have been used, such as 10 *fps* or 25 *fps* (Kong et al., 2013; Shao et al., 2016; de Fatima Galvao Rosa and Betini, 2020). Different from cameras used for estimating *f_R_* and HR, RGB digital cameras are needed for estimating SpO_2_ values since the need to work with signals with different wavelengths (see next paragraph). Considering the video resolution, the most common is 640 × 480 pixels/frame resolution, but in few studies, higher video resolutions have been employed (Cobos-Torres and Abderrahim, 2017; Casalino et al., 2020).

**Figure 5:**
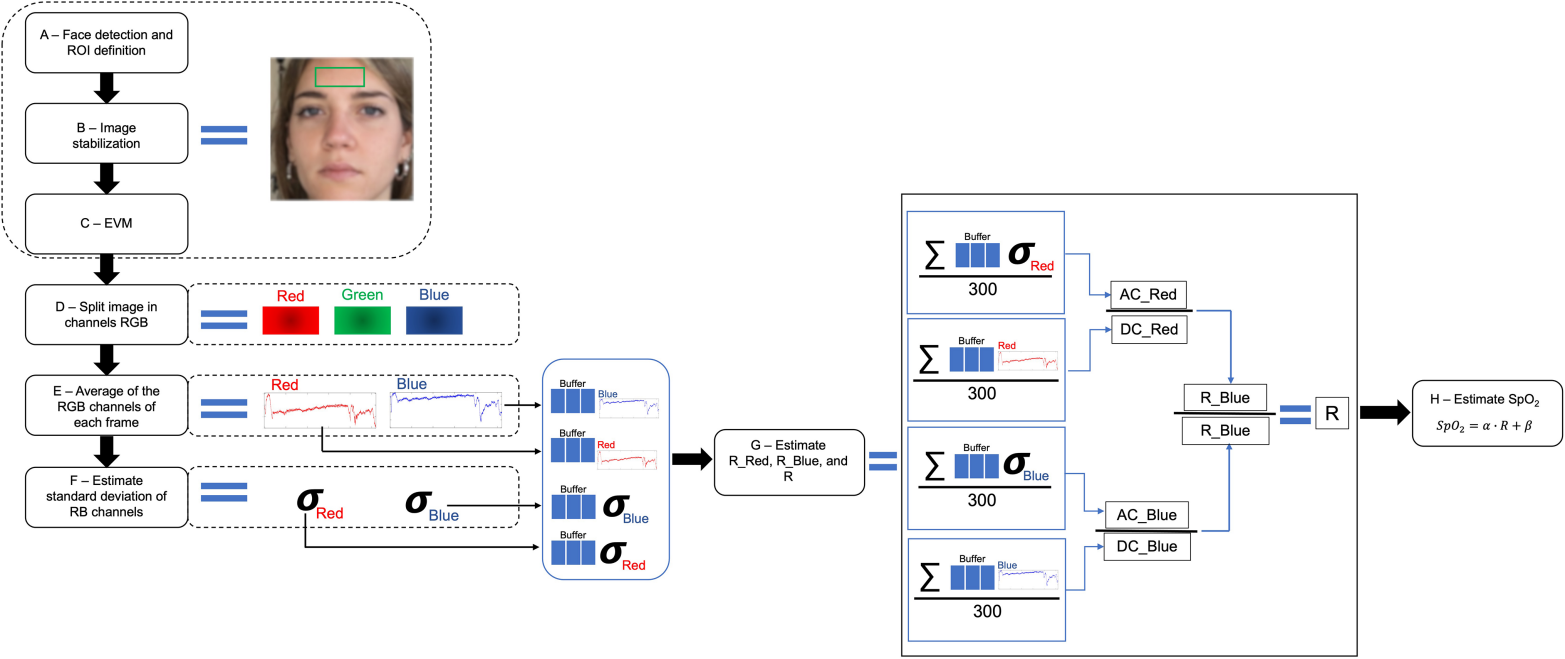
Overview of a typical framework to retrieve an estimation of SpO_2_ from the analysis of video images (adapted from Fatima Galvao Rosa and Betini, 2020).

The ROI used to extract the rPPG signal for the estimation of the SpO_2_ is the facial region. Different sites can be used as ROI, like forehead, left cheek, right cheek, or the whole face (Bal, 2015; Qayyum et al., 2017; Casalino et al., 2020; de Fatima Galvao Rosa and Betini, 2020), and in one study, the region of the lips is considered as ROI to obtain the valuable rPPG signal (Shao et al., 2016). Once a ROI has been identified, the main approach used to extract the cardiac trace is based on the analysis of the intensity changes of pixels in the selected ROI. Red and blue color channels are employed to compute the ratio of the absorbances at two typical wavelengths used in pulse oximetry (i.e., *λ*_1_ = 660 nm and *λ*_2_ = 940 nm, which corresponds to the red and IR light, respectively; Casalino et al., 2020; de Fatima Galvao Rosa and Betini, 2020). The ratio of ratio of the absorbances (RR) is computed according to the equation (1):


(1) $$RR=\frac{AC_{\lambda_{1}}/DC_{\lambda_{1}}}{AC_{\lambda_{2}}/DC_{\lambda_{2}}}=\frac{R_{\lambda_{1}}}{R_{\lambda_{2}}}=\frac{AC_{red}/DC_{red}}{AC_{blue}/DC_{blue}}=\frac{R_{red}}{R_{blue}}$$

where AC is the pulsatile component normalized by the non-pulsatile component (DC) at the wavelengths *λ*_1_ and *λ*_2_, and RR is ratio of the ratios of the absorbances at the two wavelengths (Casalino et al., 2020; de Fatima Galvao Rosa and Betini, 2020; Figure 5). The value of SpO_2_ can be estimated from RR, since there is a nearly linear relationship between them according to the equation (2) (Kong et al., 2013; Bal, 2015; Shao et al., 2016):


(2) $$SpO_{2}=\alpha \cdot RR+\beta$$

where α and β are determined by considering the linear regression for each volunteer. In particular, α represents the slope of the estimated regression line, and β is the point at which the estimated regression line intersects y (Shao et al., 2016).

Besides the methods described above, only recently the first convolutional neural network (CNN) scheme for the estimation of SpO_2_ value from a video of the palm/back side of the hand recorded with a consumer-grade RGB smartphone camera was proposed in (Mathew et al., 2021). Although the results are encouraging (see Table 3—Root Mean Square Error RMSE <3.1%), limitations related to the learning phases of the network needed for implementing this CNN scheme remain.

**Table 3:** Studies using digital cameras for estimating SpO_2_ in different fields of application.

Author, Year, Reference	Sensor, resolution, framerate	Reference system	Methods to extract the signal	Fields of application, population, scenario	Main results
Bal, 2015	Laptop camera, 640 × 480 pixels, 30 *fps*	Pulse oximeter	Skin detection	Tests in laboratory (6 seated subjects) and in PICU (3 subjects lying on the bed). Distance of about 0.5 m	*r* = 0.71 for PICU patients, and *r* > 0.81 for healthy subjects
Casalino et al., 2020	Laptop camera, 1920 × 1080 pixels, 30 *fps*	Pulse oximeter	Intensity changes of pixels	Tests in laboratory on 21 subjects. Distance of about 0.5 m	Bland–Altman MOD ± LOAs = −0.5 ± 1.95%
Cobos-Torres and Abderrahim, 2017	Webcam, 1280 × 800 pixels, 30 *fps*	Pulse oximeter	Intensity changes of pixels	Tests in laboratory on 10 subjects. Distance of about 0.5 m	Bland–Altman MOD ± LOAs = 0.3 ± 2.4%
Guazzi et al., 2015	RGB camera, 16 *fps*	Pulse oximeter	Color-based methods	Tests in laboratory on 5 subjects in a controlled hypoxic environment. Distance of about 1.5 m	Bland–Altman MOD ± LOAs = 0.01 ± 5.51%
Kong et al., 2013	Two monochrome cameras, 320 × 240 pixels, 25 *fps*	Pulse oximeter	Color-based method	Tests in laboratory on 30 subjects. Distance of about 1.5 m	Bland–Altman MOD ± LOAs = 0.4 ± 3.6%
Mathew et al., 2021	Smartphone camera, 30 *fps*	Pulse oximeter	Color-based method	Tests in laboratory on 14 subjects.	RMSE <3.1%. The results are obtained by using CNN approach.
Qayyum et al., 2017	Smartphone camera, 320 × 580 pixels, 30 *fps*	Pulse oximeter	POS	Tests in laboratory on 20 subjects.	N.A.
de Fatima Galvao Rosa and Betini, 2020; Rossol et al., 2020	Raspberry Pi camera, 30 *fps*	Pulse oximeter	Color-based method	Tests in laboratory on 9 subjects. Distance of about 0.6 m	Bland–Altman MOD ± LOAs = −0.1 ± 2.1%
Shao et al., 2016	Monochrome camera, 10 *fps*	Pulse oximeter	Color-based method	Tests in laboratory on 6 subjects	Bland–Altman MOD ± LOAs = −0.07 ± 2.6%

The sensor, resolution, and framerate of the proposed systems are specified as well as the methods used to extract the rPPG waveform. Details on the fields of application and enrolled populations, as well as details on the reference systems, are provided. The main results of each study are reported in the last column (POS, Plane orthogonal to skin; CNN, Convolutional neural network; MOD, Mean of difference; LOA, Limit of agreements; *r*, Correlation coefficient; N.A, Means not available).

Considering the fields of application, few studies tested a camera-based system for estimating SpO_2_ in clinical settings, such as in Pediatric Intensive Care Unit (i.e., PICU) or specific wards (e.g., dialysis ward). Commonly, the system involves a camera to record a video of the patients and a reference system (i.e., pulse oximeter) that allows the measurement of the ground truth values. Authors in (Bal, 2015) performed experiments in PICU settings to monitor pediatric patients. A built-in laptop camera was used to record a video of the face of the patient, and a pulse oximeter was used as a reference system. The video analysis has been carried out to extract the rPPG signal that is used to compute the value of SpO_2_ according to (2). Results show a correlation of 0.71 between the reference and the estimated values. Similarly, Tarassenko et al. tested a camera-based system by monitoring patients during a dialysis session. The rPPG signals were extracted by processing ROIs from the face of the patient minimally affected by spurious movements. SpO_2_ values were computed as in (2), and results show a close correspondence between the estimated values and the reference SpO_2_ values (Tarassenko et al., 2014). Different from the other vital signs that can be externally easily controlled by simulating elevated *f_R_* or increased HR values with exercise or stressors, SpO_2_ values can be controlled partially by carrying out hypoventilation exercises or apneas. Outside the clinical scenarios, most of the studies tested the camera-based system in laboratory environment to assess its performances and to evaluate the robustness of the system in the estimation of the parameter. Mainly, the system is composed of one RGB camera and a reference system (i.e., pulse oximeter). During the experiments, the subject is required to sit in front of the camera at a distance of about 0.5 m, remaining as motionless as possible: to evaluate the changes in SpO_2_, after normal breathing the subject is required to hold the breath for about 30 s or until he/she feels uncomfortable (i.e., during the apnea stage there is a decrease of the value of SpO_2_; Shao et al., 2016; Qayyum et al., 2017; Casalino et al., 2020; de Fatima Galvao Rosa and Betini, 2020). Table 3 summarizes the main studies dealing with SpO_2_ estimation from videos.

### Blood Pressure

The measurement of blood pressure (BP) indicates the pressure exerted by blood against the arterial wall, providing information about the blood flow during the systolic phase (i.e., contraction of the heart) and the diastolic phase (i.e., relaxation of the heart; Dias and Cunha, 2018). Normal values are in the range between 90/60 mmHg and 120/80 mmHg (systolic/diastolic); instead, higher values are diagnosed as hypertension. The cuffless estimation of BP is a very hot topic even with wearable devices (Sharma et al., 2017; Bard et al., 2019). In the last years, researchers are focusing on the estimation of BP from rPPG signal recorded through digital cameras (Jain et al., 2016; Khong et al., 2017; Luo et al., 2019; Rong and Li, 2021).

**Figure 6:**
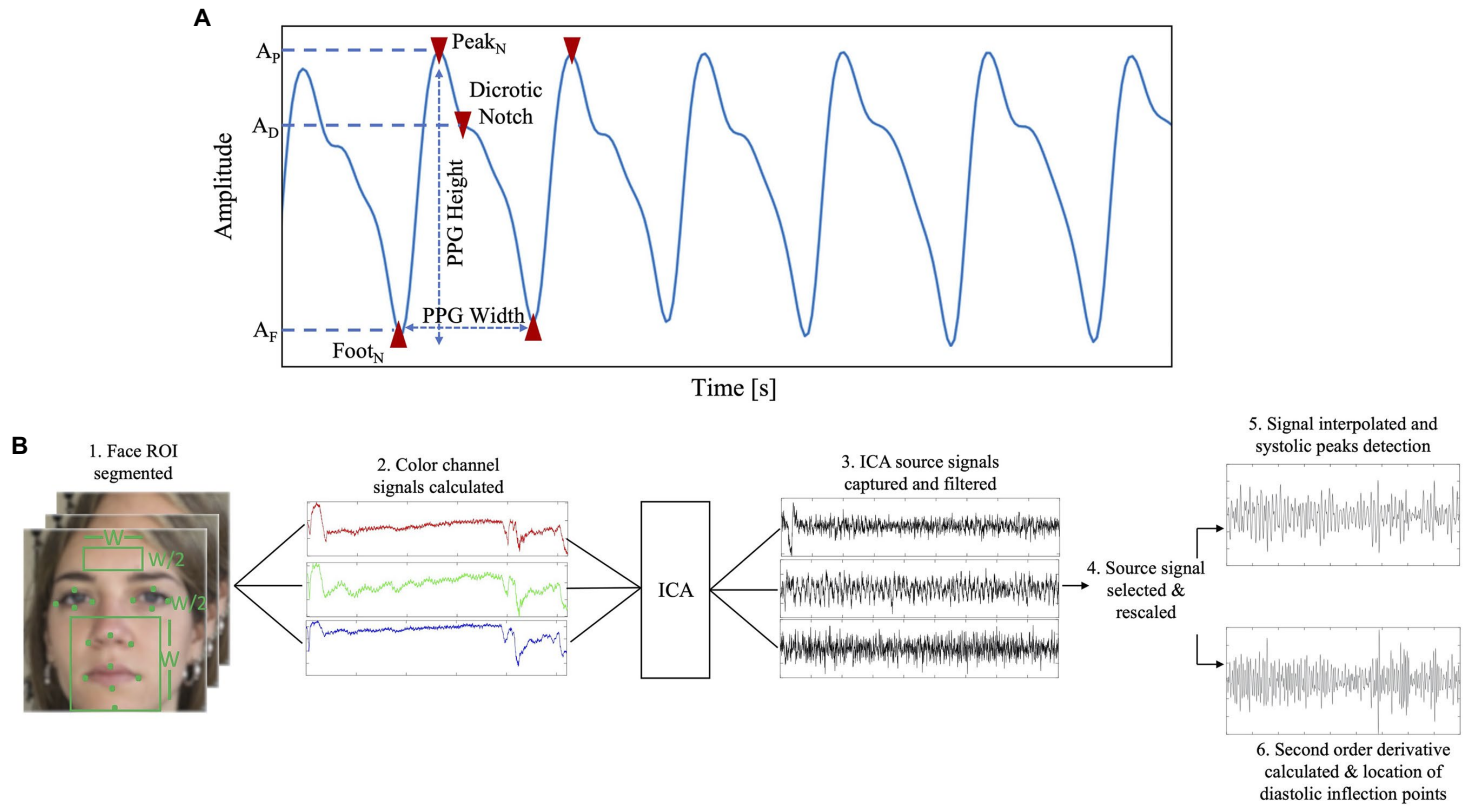
**(A)** Example of different PPG parameters which can be extracted based on the morphology of the signal (e.g., PPG width, PPG height, dicrotic notch, PPG peak, PPG foot). **(B)** Flowchart to retrieve SBP and DPB from a rPPG signal (adapted from McDuff et al., 2014).

The usual experimental setup consists of a video camera positioned in front of the user. Different distances between the camera and the user can be employed, ranging from 0.4 m to about 3 m (McDuff et al., 2014; Sugita et al., 2015; Shirbani et al., 2020). Since the measurement is based on the rPPG recordings, the same guidelines reported for the HR estimation should be followed. A framerate of 30 *fps* is the most employed since most of the cameras work on this range. However, in some studies, values of framerate higher have been employed (Jain et al., 2016; Khong et al., 2017). Considering the video resolutions, different values can be used (McDuff et al., 2014; Jain et al., 2016; Secerbegovic, 2016). Typically, the face is used as ROI and the main approach to retrieve the rPPG signal is based on the analysis of the intensity changes of pixels (Sugita et al., 2015; Khong et al., 2017). When the rPPG signal is extracted, an estimation of BP can be obtained by analyzing the morphology of the signal deriving different waveform parameters (Figure 6A), as reported in Jain et al., (2016). Authors in McDuff et al., (2014) proposed a method for the automatic identification of systolic and diastolic peaks in rPPG signals (Figure 6B) recorded by the face and found high correlation (*r* = 0.94) with signals collected from a fingertip sensor. Alternatively, pulse transit time (PTT) based parameters (Lu et al., 2020) can be used to estimate BP.

An approach to derive the values of BP is to use a physical model based on PTT, which can be obtained from the pulse wave velocity (PWV; Lu et al., 2020). PWV is related to the velocity of the pulse wave flowing in blood vessel, and according to the Moens–Korteweg equation [further details are provided in (Lu et al., 2020)], it is influenced by different vascular factors (Khong et al., 2017; Lu et al., 2020). Thus, since PTT and BP have a physical relationship through PWV according to the formula (3):


(3) $$PWV=\frac{L}{TD}=\sqrt{\frac{E_{0}he^{\alpha P}}{\rho D}}$$

where L is a length defined for TD, $E_{0}$ is the stiffness for zero pressure, *h* is the arterial wall thickness, α is a vessel parameter, *P* is the blood pressure, ρ is the density of the blood, and *D* is the diameter of the vessels (Lu et al., 2020), different mathematical models can be derived (e.g., logarithmic, linear, or quadratic). The most popular is the linear one [formula (4)]:


(4) BP=a⋅PTT+b

where *a* and *b* are different constant parameters, obtained by fitting the PTT-BP data (Lu et al., 2020). However, PTT allows only the estimation of the systolic blood pressure (SBP), and in one study, the PWV has been used to estimate SBP and DBP (diastolic blood pressure; Khong et al., 2017). However, it is worth mentioning that there are alternative methods to the above mentioned, Furthermore, in some studies Machine Learning and Deep Learning techniques are implemented to obtain an estimation of SBP and DBP (Chowdhury et al., 2020; el Hajj and Kyriacou, 2020; Rong and Li, 2021; Schrumpf et al., 2021).

To the best of our knowledge, only a few studies reported experiments in lab settings aiming at evaluating the BP estimation performances. There are no specific tests in real-life scenarios, like clinical settings. In validation studies, cuff blood pressure or invasive blood pressure medical devices were used as reference systems for SBP and DBP values. During the test, the subject is required to sit in front of the camera, facing it and remaining as motionless as possible (McDuff et al., 2014; Sugita et al., 2015; Khong et al., 2017; Shirbani et al., 2020; Rong and Li, 2021). In one study, tests have been carried out after a short exercise session (i.e., self-paced running in place for 4 min; Secerbegovic, 2016), and in another study, measurements during cognitive tasks have been performed (McDuff et al., 2014). Among others, Sugita et al. obtained BP focusing on the PTT. Three ROIs were selected to extract the rPPG signals (i.e., right palm area, forehead area, and left cheek area). PTT is obtained from the time difference between the R-peak of ECG and the minimum point of the PPG signal measured from a sensor attached to the finger, while by combing the rPPG signals of the three different ROIs the pulse propagation time difference (TD) is computed as an index equivalent to PTT. A cross-correlation coefficient has been computed between the SBP and the pulse propagation time difference (TD; Sugita et al., 2015). Authors in (Khong et al., 2017) proposed a camera-based system to estimate the values of SBP and DBP from the PWV value, computed by using the (3). Comparing the obtained results with those obtained from a reference system (i.e., upper arm blood pressure monitor), the absolute error for SBP was 4.22 ± 3.5 mmHg and for DBP is 3.24 ± 2.21 mmHg, that are acceptable error for BP (i.e., error less than 5 ± 8 mmHg as reported in ANSI/AAMI/ISO 81060-2:2013 standard). Interesting is the study of (Rong and Li, 2021), where four different regression models were implemented to find the best one for BP prediction by using Machine Learning. Results show that the best algorithm to predict BP was Support Vector Regression (SVR) and comparing the results from SVR model and the AAMI standard, a MAE of 9.97 mmHg and standard deviation (STD) of 3.35 mmHg for SBP, and a MAE of 7.59 mmHg and STD of 2.58 mmHg for DBP were obtained. These values are acceptable according to the AAMI standard. Besides the reported studies, authors in (Luo et al., 2019) used Transdermal Optical Imaging (TOI) to obtain a hemoglobin-rich signal from a video of the user’s face through ML algorithms on a large population (i.e., 1,328 normotensive adults) to estimate SBP and DBP. The implemented model allows predicting BP with a measurement error of 0.39 ± 7.30 mmHg for SBP and − 0.2 ± 6.00 mmHg for DBP. Table 4 summarizes important papers focusing on BP estimation from digital camera videos.

**Table 4:** Studies using digital cameras for estimating BP in different fields of application.

Author, Year, Reference	Sensor, resolution, framerate	Reference system	Methods to extract the signal	Fields of application, population, scenario	Main results
Jain et al., 2016	Sony camera, 1920 × 1080 pixels, 50 *fps*	Digital BP monitor	PCA	Tests in laboratory on 45 seated subjects with the eyes closed. Distance of about 0.5 m	MAE = 3.90 mmHg for systolic BP; MAE = 3.72 mmHg for diastolic BP
Khong et al., 2017	Laptop camera, 1280 × 720 pixels, 30 *fps*	Cuff-based system	Color-based method	Tests in laboratory on 45 subjects.	MAE ± STD = 4.22 ± 3.15 mmHg for SBP; MAE ± STD = 3.24 ± 2.21 mmHg for DBP
Luo et al., 2019	Smartphone camera, 1280 × 720 pixels, 30 *fps*	Cuff-based system	Color-based method (TOI)	Tests in laboratory on 1,328 normotensive adults. Distance between 0.4–0.6 m	Mean error ± STD = 0.39 ± 7.30 mmHg for SBP; Mean error ± STD = −0.20 ± 6.00 mmHg for DBP The results are obtained by using ML methods.
McDuff et al., 2014	Reflex camera, 960 × 720 pixels, 30 *fps*	Pulse oximeter	ICA	Tests in laboratory on 14 subjects at rest and during a cognitive task. Distance of about 3 m.	Quality of the rPPG waveforms by computing the Systolic-Diastolic Peak-to-peak times SD-PPTs. Correlation *r* = 0.94 between proposed and reference signal.
Rong and Li, 2021	Logitech webcam, 1280×720, 30 *fps*	Sphygmomanometer	Color-based method	Tests in laboratory on 191 subjects (normal and abnormal BP groups were included).	MAE ± STD = 9.97 ± 3.35 mmHg for SBP (Mean Error = 2.1 mmHg); MAE ± STD = 7.59 ± 2.58 mmHg for DBP (Mean Error = 0.79 mmHg). The results are obtained by using ML methods.
Secerbegovic, 2016	Digital camera, 720 × 576 pixels, 25 *fps*	Invasive arterial BP	ICA	Tests in laboratory on 3 subjects performing a baseline session and two post-exercise recovery sessions.	Mean error ± STD = 9.48 ± 7.13 mmHg for SBP. No information on DBP.
Shirbani et al., 2020	Logitech webcam, 640 × 480 pixels, 30 *fps*	Pulse oximeter	ICA	Tests in laboratory on 12 subjects in rest position. Distance between 0.4–0.6 m	Evaluation of the influence of lighting of PTT measurement: results show that PTT values decrease at higher lumens.
Sugita et al., 2015	Camera, 440 × 400 pixels, 140 *fps*	BP sensor	GC	Tests in laboratory on 20 subjects. Distance of about 1 m	Correlation coefficient r ± STD around 0.43 ± 0.30 for SBP.

The sensor, resolution and framerate of the proposed systems are specified as well as the methods used to extract the rPPG waveforms. Details on the fields of application and enrolled populations, as well as details on the reference systems are provided. The main results of each study are reported in the last column (GC, Green channel; PCA, Principal component analysis; ICA, Independent component analysis; TOI, Transdermal optical imaging; ML, Machine learning; MAE, Mean absolute error; STD, Standard deviation, N.A, Means not available).

## Conclusion

In the present review, we have provided an overview of the contactless methods for measuring vital signs based on the analysis of video-recorded through digital cameras. The work was stimulated by the wide interest in recording robust physiological signals with cameras, commonly integrated into off-the-shelf devices (e.g., laptop, smartphone, surveillance cameras, and smart TV), trying to overcome limitations and pitfalls of classical methods that require contact with the skin or dedicated hardware to perform the measurement. More deeply, the integrability of digital cameras in several devices also motivates the design of innovative products and solutions able to provide high quality of data with an “all in one” approach. For these reasons in the selection of the articles, we focused on all of those dealing with technical solutions for measuring vital signs from video images in the visible light range and applications of camera-based approaches for measuring physiological quantities in different scenarios (from clinical settings to occupational and sports fields).

The analysis of the literature reveals promising results in the monitoring of both cardiac and respiratory parameters (e.g., HR and *f_R_*) by using visible light cameras when compared to well-established medical-grade systems and wearable devices. Differently, only few studies investigated the potentialities for monitoring BP and SpO_2_, thus further validation studies are needed. Consequently, under certain experimental conditions (i.e., absence of body movements, adequate environmental light, and appropriate calibrations), the use of camera-based technologies can favor the remote monitoring of physiological quantities, that until now are measured with wearable devices or traditional hardware, with enormous potentialities in the remote personal health status assessment, clinical follow-up and in the case of integrated clinical exams (e.g., vital signs estimation during a postural evaluation with camera-based approaches; Koumbourlis, 2006; Kamelska-Sadowska et al., 2020).

Furthermore, because of the possibility to use sensors that are already available at home (e.g., video surveillance cameras, smartphones, and smart TV) or easily accessible ones (i.e., webcams), camera-based approaches for estimating vital signs could significantly influence the actual standard for vital signs monitoring of individuals under home-quarantine who need a high level of care and, in general, of those requiring continuous monitoring [e.g., after hospital discharge and during COVID-19 (Gordon et al., 2020; Massaroni et al., 2020)] with the application of a typical telemedicine approach. Moreover, monitoring the parameters reviewed in this paper could help to provide a complete representation of the clinical status of the user to the health care providers while doing televisits and teleconsultations, with a wide range of possible applications during pandemics and not. The contextual measurement of several biometrical parameters may lay the bases of a new biometrical holistic of the human body (BHOHB) approach that looks at the whole person considering their physical, emotional, and social wellbeing. As WHO reports in the Global Digital Health Strategy 2020–2025, the authors are convinced that there is space for further innovative ideas based on the reviewed technologies to address public health challenges in the post-pandemic era (WHO, 2021).

Beyond the vital parameters, camera-based systems based upon digital images analysis are still not much applied to specific domains of clinical practice. Among others, given the possibility to record the respiratory waveforms (Section “Respiratory Frequency and Other Respiratory Parameters”) and SpO_2_ values (Section “Blood Pressure”), these systems could enable the investigation of some respiratory disorders (e.g., identification of obtrusive sleep apnea; van Gastel et al., 2021) without requiring cumbersome instrumentation. By levering the multi-point measurement that can be carried out with a single uncalibrated camera or calibrated one (Heikkila and Silven, 1997; Zhang and Member, 2000; Scaramuzza et al., 2006; Urban et al., 2015), digital videos could be used for assessing respiratory biomechanics and to evaluate eventual thoraco-abdominal asynchronies and abnormalities that are pretty common in clinical practice and of interest in athletes (Black and Millard, 2001). We think that the great performances of traditional and machine learning algorithms in the estimation of cardiac-related parameters (Section “Heart Rate and Other Cardiac Parameters”) could stimulate research on the estimation of autonomic markers, including stress and wellbeing assessment from rPPG signal extracted from videos recorded in the visible light range. Moreover, with the recent advancement of deep learning, we envision blood oxygen saturation measurement will be much more reliable in the near future, with a significant positive impact on remote measurement of patient health status.

From a technical point of view, the main challenges are related to the reduction of motion artifacts and noise cancellation with the aim to improve the rPPG and motion-based respiratory signals quality and extend the usability of the camera-based even in the presence of unphysiological movements (i.e., during sports activities). At this scope, further research on torso and face tracking is suggested (Zhao et al., 2019; Liu et al., 2021). We think that a collaboration leveraging the expertise of clinicians, engineers, and data scientists could be interesting to facilitate the advancements in the application of camera-based systems methods in a wide range of populations and everyday life and to push innovation toward “all in one” products and solutions.

## Author Contributions

NM, CM, ES, and SS wrote and edited the manuscript. NM, CM, ES, EV, DA, FBo, FBu, and SS contributed to the editing and revising the manuscript. All authors contributed to the article and approved the submitted version.

## Funding

This work was carried out in the framework of the project titled “Sviluppo di sistemi di misura e modelli per la stima senza contatto di parametri fisiologici,” POR Lazio FSE 2014/2020 (CUP code no. F87C21000190009, Project ID 23562).

## Conflict of Interest

FBo, DA, EV, and FBu are employed by the company BHOHB S.r.l.

The remaining authors declare that the research was conducted in the absence of any commercial or financial relationships that could be construed as a potential conflict of interest.

## Publisher’s Note

All claims expressed in this article are solely those of the authors and do not necessarily represent those of their affiliated organizations, or those of the publisher, the editors and the reviewers. Any product that may be evaluated in this article, or claim that may be made by its manufacturer, is not guaranteed or endorsed by the publisher.

## References

[ref1] Aarts L. A. M. Jeanne V. Cleary J. P. Lieber C. Nelson J. S. Bambang Oetomo S.. (2013). Non-contact heart rate monitoring utilizing camera photoplethysmography in the neonatal intensive care unit - A pilot study. Early Hum. Dev. 89, 943–948. doi: 10.1016/j.earlhumdev.2013.09.016, PMID: 24135159

[ref2] Abdi H. Williams L. J. (2010). Principal component analysis. Wiley Interdiscip. Rev. Comput. Stat. 2, 433–459. doi: 10.1002/wics.101

[ref3] Alinovi D. Ferrari G. Pisani F. Raheli R. (2018). Respiratory rate monitoring by video processing using local motion magnification. *European Signal Processing Conference*; September, 2018, 1780–1784.

[ref4] Al-naji A. Chahl J. (2017). Contactless cardiac activity detection based on head motion magnification. Inter. J. Image Graphics 17:7500012. doi: 10.1142/S0219467817500012

[ref5] Al-Naji A. Gibson K. Lee S. H. Chahl J. (2017). Monitoring of cardiorespiratory signal: principles of remote measurements and review of methods. IEEE Access 5, 15776–15790. doi: 10.1109/ACCESS.2017.2735419

[ref6] Antognoli L. Marchionni P. Spinsante S. Nobile S. Carnielli V. P. Scalise L. (2019). “Enanced video heart rate and respiratory rate evaluation: standard multiparameter monitor vs clinical confrontation in newborn patients. Medical measurements and applications,” in *MeMeA 2019 - Symposium Proceedings*. June 26–28, 2019; Instabul, Turkey; 1–5.

[ref7] Antognoli L. Moccia S. Migliorelli L. Casaccia S. Scalise L. Frontoni E. (2020). Heartbeat detection by laser doppler vibrometry and machine learning. Sensors 20, 1–18. doi: 10.3390/s20185362 PMC7571227 32962134

[ref8] Bal U. (2015). Non-contact estimation of heart rate and oxygen saturation using ambient light. Biomed. Opt. Express 6:86. doi: 10.1364/boe.6.000086 25657877 PMC4317113

[ref9] Balakrishnan G. Durand F. Guttag J. (2013). “Detecting pulse from head motions in video.” in *Proceedings of the IEEE Conference on Computer Vision and Pattern Recognition*. June 23–28, 2013; Portland, Oregon, USA; 3430–3437.

[ref10] Bard D. M. Joseph J. I. van Helmond N. (2019). Cuff-less methods for blood pressure Telemonitoring. Front. Cardiovascular Med. 6, 1–7. doi: 10.3389/fcvm.2019.00040 PMC6502966 31157236

[ref11] Bartula M. Tigges T. Muehlsteff J. (2013). “Camera-based system for contactless monitoring of respiration. Conference proceedings: annual international conference of the IEEE engineering in medicine and biology society.” in *IEEE Engineering in Medicine and Biology society. Annual Conference*. July 3–7, 2013; Osaka, Japan; 2672-2675. 10.1109/EMBC.2013.6610090 24110277

[ref12] Béres S. Hejjel L. (2021). The minimal sampling frequency of the photoplethysmogram for accurate pulse rate variability parameters in healthy volunteers. Biomed. Signal Process. Control 68:102589. doi: 10.1016/j.bspc.2021.102589

[ref13] Bernardi L. Porta C. Gabutti A. Spicuzza L. Sleight P. (2001). Modulatory effects of respiration. Auton. Neurosci. Basic Clinic. 90, 47–56. doi: 10.1016/S1566-0702(01)00267-3 11485292

[ref14] Black A. M. S. Millard R. K. (2001). Assessing thoracoabdominal asynchrony. Clin. Physiol. 21, 383–385. doi: 10.1046/j.1365-2281.2001.00325.x, PMID: 11380539

[ref15] Boric-Lubecke O. Lubecke V. M. Mostafanezhad I. Park B.-K. Massagram W. Jokanovic B. (2009). Doppler radar architectures and signal processing for heart rate extraction. Mikrotalasna revija 15, 12–17.

[ref107] Brieva J. Moya-Albor E. Rivas-Scott O. Ponce H. (2018). “Non-contact breathing rate monitoring system based on a Hermite video magnification technique.” in 14th International Symposium on Medical Information Processing and Analysis. *Vol. 10975*. October 24–26, 2018; Mazatlán, Mexico; International Society for Optics and Photonics; 1097504.

[ref16] Casalino G. Castellano G. Zaza G. (2020). A mHealth solution for contact-less self-monitoring of blood oxygen saturation. *Proceedings - IEEE symposium on computers and communications*; July, 2020.

[ref17] Cennini G. Arguel J. Akşit K. van Leest A. (2010). Heart rate monitoring via remote photoplethysmography with motion artifacts reduction. Opt. Express 18:4867. doi: 10.1364/oe.18.004867 20389499

[ref18] Chaichulee S. Villarroel M. Jorge J. O. Arteta C. McCormick K. Zisserman A.. (2019). Cardio-respiratory signal extraction from video camera data for continuous non-contact vital sign monitoring using deep learning. Physiol. Meas. 40:ab525c. doi: 10.1088/1361-6579/ab525c PMC7655150 31661680

[ref19] Chatterjee A. Prathosh A. P. Praveena P. (2016). “Real-time respiration rate measurement from thoracoabdominal movement with a consumer grade camera.” in *Proceedings of the Annual International Conference of the IEEE Engineering in Medicine and Biology Society, EMBS*. August 16–20, 2016; Orlando, Florida; 2708–2711. 10.1109/EMBC.2016.7591289 28268880

[ref20] Chen W. (2018). “DeepPhys: video-based physiological measurement using convolutional attention networks.” in *Proceedings of the European Conference on Computer Vision (ECCV)*. September 8–14, 2018; Munich, Germany.

[ref21] Chen L. Liu N. Hu M. Zhai G. (2019a). Rgb-thermal imaging system collaborated with marker tracking for remote breathing rate measurement. *2019 IEEE International Conference on Visual Communications and Image Processing*, *VCIP 2019*. December 1–4, 2019; Sydney, Australia. 2019–2022.

[ref22] Chen M. Zhu Q. Zhang H. Wu M. Wang Q. (2019b). Respiratory rate estimation from face videos. *2019 IEEE EMBS International Conference on Biomedical and Health Informatics, BHI 2019 – Proceedings*; 3–6.

[ref23] Cheng X. Yang B. Olofsson T. Liu G. Li H. (2017). A pilot study of online non-invasive measuring technology based on video magni fi cation to determine skin temperature. Build. Environ. 121, 1–10. doi: 10.1016/j.buildenv.2017.05.021

[ref24] Choi A. Shin H. (2017). Photoplethysmography sampling frequency: pilot assessment of how low can we go to analyze pulse rate variability with reliability? Physiol. Meas. 38, 586–600. doi: 10.1088/1361-6579/aa5efa 28169836

[ref25] Chowdhury M. H. Shuzan M. N. I. Chowdhury M. E. H. Mahbub Z. B. Monir Uddin M. Khandakar A.. (2020). Estimating blood pressure from the photoplethysmogram signal and demographic features using machine learning techniques. Sensors 20:3127. doi: 10.3390/s20113127 PMC7309072 32492902

[ref26] Chung E. Chen G. Alexander B. Cannesson M. (2013). Non-invasive continuous blood pressure monitoring: A review of current applications. Front. Med. China 7, 91–101. doi: 10.1007/s11684-013-0239-5, PMID: 23345112

[ref27] Cobos-Torres J. C. Abderrahim M. (2017). Simple measurement of pulse oximetry using a standard color camera. *2017 40th International Conference on Telecommunications and Signal Processing*; January, 2017, 452–455.

[ref28] Cretikos M. A. Bellomo R. Hillman K. Chen J. Finfer S. Flabouris A. (2008). Respiratory rate: the neglected vital sign. Med. J. Aust. 188, 657–659. doi: 10.5694/j.1326-5377.2008.tb01825.x 18513176

[ref29] Davila M. I. Lewis G. F. Porges S. W. (2017). The PhysioCam: A novel non-contact sensor to measure heart rate variability in clinical and field applications. Front. Public Health 5, 1–14. doi: 10.3389/fpubh.2017.00300 29214150 PMC5702637

[ref30] de Fatima Galvao Rosa A. Betini R. C. (2020). Noncontact SpO_2_ measurement using Eulerian video magnification. IEEE Trans. Instrum. Meas. 69, 2120–2130. doi: 10.1109/TIM.2019.2920183

[ref31] de Groote A. Wantier M. Cheron G. Estenne M. Paiva M. (1997). Chest wall motion during tidal breathing. J. Appl. Physiol. 83, 1531–1537. doi: 10.1152/jappl.1997.83.5.1531 9375316

[ref32] de Haan G. Jeanne V. (2013). Robust pulse rate from chrominance-based rPPG. IEEE Trans. Biomed. Eng. 60, 2878–2886. doi: 10.1109/TBME.2013.2266196, PMID: 23744659

[ref33] Dias D. Cunha J. P. S. (2018). Wearable health devices—vital sign monitoring, systems and technologies. Sensors 18:2414. doi: 10.3390/s18082414 PMC6111409 30044415

[ref34] el Hajj C. Kyriacou P. A. (2020). “Cuffless and continuous blood pressure estimation from PPG signals using recurrent neural networks.” in *Proceedings of the Annual International Conference of the IEEE Engineering in Medicine and Biology Society, EMBS (Institute of Electrical and Electronics Engineers Inc.)*. July 20–24, 2020; Montreal, Canada (Virtual); 4269–4272. 10.1109/EMBC44109.2020.9175699 33018939

[ref35] Elliott M. Coventry A. (2012). Signs of patient monitoring. Br. J. Nurs. 21, 621–625. doi: 10.12968/bjon.2012.21.10.621 22875303

[ref36] Favilla R. Zuccala V. C. Coppini G. (2019). Heart rate and heart rate variability from Single-Channel video and ICA integration of multiple signals. IEEE J. Biomed. Health Inform. 23, 2398–2408. doi: 10.1109/JBHI.2018.2880097, PMID: 30418892

[ref37] Foo J. Y. A. Chua K. P. Tan X. J. A. (2013). Clinical applications and issues of oxygen saturation level measurements obtained from peripheral sites. J. Med. Eng. Technol. 37, 388–395. doi: 10.3109/03091902.2013.816380, PMID: 23859608

[ref38] Ganfure G. O. (2019). Using video stream for continuous monitoring of breathing rate for general setting. SIViP 13, 1395–1403. doi: 10.1007/s11760-019-01486-5

[ref39] Garbey M. Sun N. Merla A. Pavlidis I. (2004). Contact-free measurement of cardiac pulse based on the analysis of thermal imagery 1 Department of Computer Science Technical Report Number UH-CS-04-08.54, 1418–1426. 10.1109/TBME.2007.891930 17694862

[ref40] Gordon W. J. Henderson D. Desharone A. Fisher H. N. Judge J. Levine D. M.. (2020). Remote patient monitoring program for hospital discharged COVID-19 patients. Appl. Clin. Inform. 11, 792–801. doi: 10.1055/s-0040-1721039, PMID: 33241547 PMC7688410

[ref41] Grassmann M. Vlemincx E. von Leupoldt A. Mittelstädt J. M. van den Bergh O. (2016). Respiratory changes in response to cognitive load: A systematic review. Neural Plast. 2016, 1–16. doi: 10.1155/2016/8146809, PMID: 27403347 PMC4923594

[ref42] Guazzi A. R. Villarroel M. Jorge J. Daly J. Frise M. C. Robbins P. A.. (2015). Non-contact measurement of oxygen saturation with an RGB camera. Biomed. Opt. Express 6:3320. doi: 10.1364/boe.6.003320 26417504 PMC4574660

[ref43] Gudi A. Bittner M. van Gemert J. (2020). Real-time webcam heart-rate and variability estimation with clean ground truth for evaluation. Appl. Sci. 10, 1–24. doi: 10.3390/app10238630

[ref44] Guo Z. Wang Z. J. Shen Z. (2014). Physiological parameter monitoring of drivers based on video data and independent vector analysis. *ICASSP, IEEE International Conference on Acoustics, Speech and Signal Processing – Proceedings*; 4374–4378.

[ref45] Habib J. Baetz L. Satiani B. (2012). Assessment of collateral circulation to the hand prior to radial artery harvest. Vascular Med. 17, 352–361. doi: 10.1177/1358863X12451514, PMID: 22814998

[ref46] Hall J. E. Guyton A. C. (2011). Guyton and Hall Textbook of Medical Physiology. Elsevier Health Sciences. New York.

[ref47] Hall T. Lie D. Y. C. Nguyen T. Q. Mayeda J. C. Lie P. E. Lopez J.. (2017). Non-contact sensor for long-term continuous vital signs monitoring: A review on intelligent phased-array doppler sensor design. Sensors 17, 1–20. doi: 10.3390/s17112632, PMID: 29140281 PMC5713025

[ref48] Han B. Ivanov K. Yan Y. Wang L. (2015). “Exploration of the optimal skin-camera distance for facial photoplethysmographic imaging measurement using cameras of different types.” in *MOBIHEALTH 2015 - 5th EAI International Conference on Wireless Mobile Communication and Healthcare - Transforming Healthcare through Innovations in Mobile and Wireless Technologies*. October 14–16, 2015; London, Great Britain.

[ref49] Harford M. Catherall J. Gerry S. Young J. D. Watkinson P. (2019). Availability and performance of image-based, non-contact methods of monitoring heart rate, blood pressure, respiratory rate, and oxygen saturation: A systematic review. Physiol. Meas. 40:06TR01. doi: 10.1088/1361-6579/ab1f1d, PMID: 31051494

[ref50] Hassan M. A. Malik A. S. Fofi D. Saad N. Karasfi B. Ali Y. S.. (2017a). Heart rate estimation using facial video: a review. Biomed. Signal Proces. Control 38, 346–360. doi: 10.1016/j.bspc.2017.07.004

[ref51] Hassan M. A. Malik A. S. Fofi D. Saad N. Meriaudeau F. (2017b). Novel health monitoring method using an RGB camera. Biomed. Opt. Express 8:4838. doi: 10.1364/boe.8.004838 29188085 PMC5695935

[ref52] Heikkila J. Silven O. (1997). “A four-step camera calibration procedure with implicit image correction.” in *Proceedings of IEEE Computer Society Conference on Computer Vision and Pattern Recognition*; 1106–1112.

[ref53] Huan Qi Z. J. W. (2015). “Non-contact driver cardiac physiological monitoring using video data.” in *2015 IEEE China Summit and International Conference on Signal and Information Processing*. July 12–15, 2015; Chengdu, China; 6–10.

[ref54] Jain M. Deb S. Subramanyam A. V. (2016). “Face video based touchless blood pressure and heart rate estimation.” in *2016 IEEE 18th International Workshop on Multimedia Signal Processing, MMSP 2016*. September 21–23, 2016; Montreal, Canada.

[ref55] Janssen R. Wang W. Moço A. de Haan G. (2015). Video-based respiration monitoring with automatic region of interest detection. Physiol. Meas. 37, 100–114. doi: 10.1088/0967-3334/37/1/100, PMID: 26640970

[ref56] Jorge J. Villarroel M. Chaichulee S. Guazzi A. Davis S. Green G.. (2017). “Non-contact monitoring of respiration in the neonatal intensive care unit.” in *Proceedings - 12th IEEE International Conference on Automatic Face and Gesture Recognition, FG 2017 - 1st International Workshop on Adaptive Shot Learning for Gesture Understanding and Production, ASL4GUP 2017, Biometrics in the Wild Bwild 2017, Heteroge*. May 30–June 3, 2017; Washington, DC, USA; 286–293.

[ref57] Kamelska-Sadowska A. M. Protasiewicz-Fałdowska H. Zaborowska-Sapeta K. Nowakowski J. J. Kowalski I. (2020). The SpinalMeter biometrical assessment to improve posture diagnosis in school-age girls: A validation study. Polish Annals Med. 27, 138–146. doi: 10.29089/2020.20.00126

[ref58] Karlen W. Garde A. Myers D. Scheffer C. Ansermino J. M. Dumont G. A. (2015). Estimation of respiratory rate from Photoplethysmographic imaging videos compared to pulse Oximetry. IEEE J. Biomed. Health Inform. 19, 1331–1338. doi: 10.1109/JBHI.2015.2429746, PMID: 25955999

[ref59] Kawachi I. Sparrow D. Vokonas P. S. Weiss S. T. (1995). Decreased heart rate variability in men with phobic anxiety (data from the normative aging study). Am. J. Cardiol. 75, 882–885. doi: 10.1016/S0002-9149(99)80680-8, PMID: 7732994

[ref60] Kebe M. Gadhafi R. Mohammad B. Sanduleanu M. Saleh H. Al-qutayri M. (2020). Human vital signs detection methods and potential using radars: A review. Sensors 20:1454. doi: 10.3390/s20051454, PMID: 32155838 PMC7085680

[ref61] Khanam F. T. Z. Al-Naji A. Chahl J. (2019). Remote monitoring of vital signs in diverse non-clinical and clinical scenarios using computer vision systems: A review. Appl. Sci. 9:4474. doi: 10.3390/app9204474

[ref62] Khong W. L. Rao N. S. V. K. Mariappan M. (2017). Blood pressure measurements using non-contact video imaging techniques. *Proceedings - 2017 IEEE 2nd International Conference on Automatic Control and Intelligent Systems, I2CACIS 2017*; December, 2017, 35–40.

[ref63] Kong L. Zhao Y. Dong L. Jian Y. Jin X. Li B.. (2013). Non-contact detection of oxygen saturation based on visible light imaging device using ambient light. Opt. Express 21:17464. doi: 10.1364/oe.21.017464 23938616

[ref64] Koumbourlis A. C. (2006). Scoliosis and the respiratory system. Paediatr. Respir. Rev. 7, 152–160. doi: 10.1016/j.prrv.2006.04.009 16765303

[ref65] Kranjec J. Beguš S. Geršak G. Drnovšek J. (2014). Non-contact heart rate and heart rate variability measurements: a review. Biomed. Signal Proces. Control 13, 102–112. doi: 10.1016/j.bspc.2014.03.004

[ref66] Krolak A. (2017). “Influence of Skin Tone on Efficiency of Vision-Based Heart Rate Estimation.” in *Polish Conference on Biocybernetics and Biomedical Engineering*. September 20–22, 2017; Krakow, Poland; 44–55.

[ref67] Kuo J. Koppel S. Charlton J. L. Rudin-Brown C. M. (2015). Evaluation of a video-based measure of driver heart rate. J. Saf. Res. 54, 55.e29–55.e59. doi: 10.1016/j.jsr.2015.06.009, PMID: 26403902

[ref68] Kwon S. Kim H. Park K. S. (2012). Validation of heart rate extraction using video imaging on a built-in camera system of a smartphone. *Proceedings of the Annual International Conference of the IEEE Engineering in Medicine and Biology Society, EMBS*, 2174–2177. 10.1109/EMBC.2012.6346392 23366353

[ref69] Lamba P. S. Virmani D. (2020). Contactless heart rate estimation from face videos. J. Stat. Manag. Syst. 23, 1275–1284. doi: 10.1080/09720510.2020.1799584

[ref70] Lewandowska M. Nowak J. (2012). Measuring pulse rate with a webcam. J. Med. Imaging Health Inform. 2, 87–92. doi: 10.1166/jmihi.2012.1064

[ref71] Liu I. Ni S. Peng K. (2020). Enhancing the robustness of smartphone photoplethysmography: A signal quality index approach. Sensors 20:1923. doi: 10.3390/s20071923, PMID: 32235543 PMC7181214

[ref72] Liu Y. Qin B. Li R. Li X. Huang A. Liu H.. (2021). Motion-robust multimodal heart rate estimation using BCG fused remote-PPG with deep facial ROI tracker and pose constrained Kalman filter. IEEE Trans. Instrum. Meas. 70, 1–5. doi: 10.1109/TIM.2021.306057233776080

[ref73] Liu C. Yang Y. Tsow F. Shao D. Tao N. (2017). Noncontact spirometry with a webcam. J. Biomed. Opt. 22:057002. doi: 10.1117/1.jbo.22.5.057002, PMID: 28514470 PMC5435829

[ref74] Longin E. Schaible T. Lenz T. König S. (2005). Short term heart rate variability in healthy neonates: normative data and physiological observations. Early Hum. Dev. 81, 663–671. doi: 10.1016/j.earlhumdev.2005.03.015, PMID: 16046085

[ref75] Loughlin P. C. Sebat F. Kellett J. G. (2018). Respiratory rate: The forgotten vital sign—make it count! Jt. Comm. J. Qual. Patient Saf. 44, 494–499. doi: 10.1016/j.jcjq.2018.04.014, PMID: 30071969

[ref76] Lu C. H. Low J. H. Tuan C. C. (2018). “Non-contact pulse rate measurement of hand and wrist using RGB camera.” in *International Conference on Broadband and Wireless Computing, Communication and Applications*. October 27–29, 2018; Taichung, Taiwan.

[ref77] Lu Y. Wang C. Meng M. Q. H. (2020). “Video-based contactless blood pressure estimation: A review.” in *2020 IEEE International Conference on Real-Time Computing and Robotics, RCAR 2020*. September 28–29, 2020; Virtual event; 62–67.

[ref78] Luo H. Yang D. Barszczyk A. Vempala N. Wei J. Wu S. J.. (2019). Smartphone-based blood pressure measurement using transdermal optical imaging technology. Circ. Cardiovascular Imaging 12:e008857. doi: 10.1161/CIRCIMAGING.119.008857 31382766

[ref79] Maji S. Massaroni C. Schena E. Silvestri S. (2020). “Contactless heart rate monitoring using A standard RGB camera.” in *2020 IEEE International Workshop on Metrology for Industry 4.0 and IoT, MetroInd 4.0 and IoT 2020 - Proceedings*. June 3–5, 2020; Virtual event; 729–733.

[ref80] Massaroni C. Lo Presti D. Formica D. Silvestri S. Schena E. (2019a). Non-contact monitoring of breathing pattern and respiratory rate via rgb signal measurement. Sensor 19, 1–15. doi: 10.3390/s19122758, PMID: 31248200 PMC6631485

[ref81] Massaroni C. Lopes D. S. Lo Presti D. Schena E. Silvestri S. (2018a). Contactless monitoring of breathing patterns and respiratory rate at the pit of the neck: A single camera approach. Sensors 2018, 1–13. doi: 10.1155/2018/4567213

[ref82] Massaroni C. Nicolò A. Lo Presti D. Sacchetti M. Silvestri S. Schena E. (2019b). Contact-based methods for measuring respiratory rate. Sensor 19, 1–47. doi: 10.3390/s19040908, PMID: 30795595 PMC6413190

[ref83] Massaroni C. Nicolo A. Sacchetti M. Schena E. (2021). Contactless methods For measuring respiratory rate: a review. IEEE Sens. J. 21, 12821–12839. doi: 10.1109/jsen.2020.3023486

[ref84] Massaroni C. Nicolò A. Schena E. Sacchetti M. (2020). Remote respiratory monitoring in the time of COVID-19. Front. Physiol. 11, 1–4. doi: 10.3389/fphys.2020.00635, PMID: 32574240 PMC7274133

[ref85] Massaroni C. Schena E. Silvestri S. Maji S. (2019c). “Comparison of two methods for estimating respiratory waveforms from videos without contact.” in *Medical Measurements and Applications, MeMeA 2019 - Symposium Proceedings*. June 26–28, 2019; Instabul, Turkey.

[ref86] Massaroni C. Schena E. Silvestri S. Taffoni F. Merone M. (2018b). “Measurement system based on RBG camera signal for contactless breathing pattern and respiratory rate monitoring.” in *MeMeA 2018–2018 IEEE International Symposium on Medical Measurements and Applications, Proceedings*. June 11–13, 2018; Rome, Italy.

[ref87] Massaroni C. Senesi G. Schena E. Silvestri S. (2017). Analysis of breathing via optoelectronic systems: comparison of four methods for computing breathing volumes and thoraco-abdominal motion pattern. Comput. Methods Biomech. Biomed. Engin. 20, 1678–1689. doi: 10.1080/10255842.2017.1406081, PMID: 29164909

[ref88] Mateu-Mateus M. Guede-Fernandez F. Garcia-Gonzalez M. A. Ramos-Castro J. J. Fernandez-Chimeno M. (2020). Camera-based method for respiratory rhythm extraction from a lateral perspective. IEEE Access 8, 154924–154939. doi: 10.1109/ACCESS.2020.3018616

[ref89] Mateu-Mateus M. Guede-Fernández F. Rodriguez-Ibáñez N. García-González M. A. Ramos-Castro J. Fernández-Chimeno M. (2021). A non-contact camera-based method for respiratory rhythm extraction. Biomed. Signal Process. Control 66:102443. doi: 10.1016/j.bspc.2021.102443

[ref90] Mathew J. Tian X. Wu M. Wong C.-W. (2021). Remote Blood Oxygen Estimation From Videos Using Neural Networks. *arXiv*. [ahead of Print] 10.1109/JBHI.2023.3236631 PMC10472532 37018728

[ref91] Mcduff D. J. Blackford E. B. Estepp J. R. (2017). Fusing Partial Camera Signals for Non-Contact Pulse Rate Variability Measurement. IEEE 65, 1725–1739. doi: 10.1109/TBME.2017.2771518 29989930

[ref92] McDuff D. Gontarek S. Picard R. W. (2014). Remote detection of photoplethysmographic systolic and diastolic peaks using a digital camera. IEEE Trans. Biomed. Eng. 61, 2948–2954. doi: 10.1109/TBME.2014.2340991, PMID: 25073159

[ref93] McDuff D. J. Hernandez J. Gontarek S. Picard R. W. (2016). “COGCAM: contact-free measurement of cognitive stress during computer tasks with a digital camera.” in *Conference on Human Factors in Computing Systems - Proceedings*. May 7–12, 2016; Conference on Human Factors in Computing Systems–Proceeding, California, USA; 4000–4004.

[ref94] Mehta A. D. Sharma H. (2020). “Tracking nostril movement in facial video for respiratory rate estimation.” in *2020 11th International Conference on Computing, Communication and Networking Technologies, ICCCNT 2020*. July 1–3, 2020; Kharagpur, India.

[ref95] Mestha L. K. Kyal S. Xu B. Lewis L. E. Kumar V. (2014). “Towards continuous monitoring of pulse rate in neonatal intensive care unit with a webcam.” in *2014 36th Annual International Conference of the IEEE Engineering in Medicine and Biology Society, EMBC 2014*. August 26 – 30, 2014; Chicago, USA; 3817–3820. 10.1109/EMBC.2014.6944455 25570823

[ref96] Morbiducci U. Scalise L. de Melis M. Grigioni M. (2007). Optical vibrocardiography: A novel tool for the optical monitoring of cardiac activity. Ann. Biomed. Eng. 35, 45–58. doi: 10.1007/s10439-006-9202-9 17082980

[ref97] Nguyen J. Duong H. (2020). Anatomy, shoulder and upper limb, hand arteries. StatPearls. Available at: http://www.ncbi.nlm.nih.gov/pubmed/31536192 (Accessed October 11, 2021). 31536192

[ref98] Ni A. Azarang A. Kehtarnavaz N. (2021). A review of deep learning-based contactless heart rate measurement methods. Sensors 21:3719. doi: 10.3390/s21113719, PMID: 34071736 PMC8198867

[ref99] Nicolò A. Massaroni C. Schena E. Sacchetti M. (2020). The importance of respiratory rate monitoring: From healthcare to sport and exercise. Sensors 20, 1–45. doi: 10.3390/s20216396, PMID: 33182463 PMC7665156

[ref100] O’Brien L. M. Stebbens V. A. Poets C. F. Heycock E. G. Southall D. P. (2000). Oxygen saturation during the first 24 hours of life. Arch. Dis. Child. Fetal Neonatal Ed. 83, 35F–38F. doi: 10.1136/fn.83.1.f35, PMID: 10873169 PMC1721111

[ref101] Paul M. Karthik S. Joseph J. Sivaprakasam M. Kumutha J. Leonhardt S.. (2020). Non-contact sensing of neonatal pulse rate using camera-based imaging: A clinical feasibility study. Physiol. Meas. 41:024001. doi: 10.1088/1361-6579/ab755c, PMID: 32148333

[ref102] Poh M.-Z. McDuff D. J. Picard R. W. (2010). Non-contact, automated cardiac pulse measurements using video imaging and blind source separation. Opt. Express 18:10762. doi: 10.1364/oe.18.010762 20588929

[ref103] Poh M. Z. McDuff D. J. Picard R. W. (2011). Advancements in noncontact, multiparameter physiological measurements using a webcam. IEEE Trans. Biomed. Eng. 58, 7–11. doi: 10.1109/TBME.2010.2086456, PMID: 20952328

[ref104] Qayyum A. Malik A. S. Shuaibu A. N. Nasir N. (2017). “Estimation of non-contact smartphone video-based vital sign monitoring using filtering and standard color conversion techniques.” in *2017 IEEE Life Sciences Conference, LSC 2017*. December 13–15, 2017; Sydney, Australia; 202–205.

[ref105] Rasche S. Trumpp A. Waldow T. Gaetjen F. Plotze K. Wedekind D.. (2016). Camera-based photoplethysmography in critical care patients. Clin. Hemorheol. Microcirc. 64, 77–90. doi: 10.3233/CH-162048 26890242

[ref106] Reyes B. A. Reljin N. Kong Y. Nam Y. Chon K. H. (2017). Tidal volume and instantaneous respiration rate estimation using a volumetric surrogate signal acquired via a smartphone camera. IEEE J. Biomed. Health Inform. 21, 764–777. doi: 10.1109/JBHI.2016.2532876, PMID: 26915142

[ref108] Romano C. Schena E. Silvestri S. Massaroni C. (2021). Non-contact respiratory monitoring using an RGB camera for real-world applications. Sensors 21:5126. doi: 10.3390/s21155126, PMID: 34372363 PMC8347288

[ref109] Rong M. Li K. (2021). A blood pressure prediction method based on imaging Photoplethysmography in combination with machine learning. Biomed. Signal Proces. Control 64:102328. doi: 10.1016/j.bspc.2020.102328

[ref110] Rossol S. L. Yang K. Toney-noland C. Bergin J. Basavaraju C. Kumar P.. (2020). Non-contact video-based neonatal respiratory monitoring. MDPI 7:100171. doi: 10.3390/children7100171 PMC7600716 33036226

[ref111] Rouast P. Adam M. T. P. Chiong R. Cornforth D. Lux E. (2018). Remote heart rate measurement using low-cost RGB face video: a technical literature review. Front. Comp. Sci. 12, 858–872. doi: 10.1007/s11704-016-6243-6

[ref112] Sanyal S. Nundy K. K. (2018). Algorithms for monitoring heart rate and respiratory rate From the video of a User’s Face. IEEE J. Trans. Eng. Health Med. 6, 1–11. doi: 10.1109/JTEHM.2018.2818687 PMC5957265 29805920

[ref113] Scalise L. Bernacchia N. Ercoli I. Marchionni P. (2012). Heart rate measurement in neonatal patients using a webcamera. *MeMeA 2012–2012 IEEE Symposium on Medical Measurements and Applications, Proceedings*, 6–9. doi: 10.1109/MeMeA.2012.6226654.

[ref114] Scaramuzza D. Martinelli A. Siegwart R. Scaramuzza D. Martinelli A. Siegwart R. (2006). Cameras to cite this version: A toolbox for easily calibrating omnidirectional cameras. Iros.

[ref115] Schena E. Massaroni C. Saccomandi P. Cecchini S. (2015). Flow measurement in mechanical ventilation: A review. Med. Eng. Phys. 37, 257–264. doi: 10.1016/j.medengphy.2015.01.010, PMID: 25659299

[ref116] Schrumpf F. Frenzel P. Aust C. Osterhoff G. Fuchs M. (2021). Assessment of non-invasive blood pressure prediction from ppg and rppg signals using deep learning. Sensors 21:6022. doi: 10.3390/s21186022, PMID: 34577227 PMC8472879

[ref117] Schrumpf F. Monch C. Bausch G. Fuchs M. (2019). “Exploiting weak head movements for camera-based respiration detection.” in *Proceedings of the Annual International Conference of the IEEE Engineering in Medicine and Biology Society, EMBS*. July 23–27, 2019; Berlin, Germany; 6059–6062. 10.1109/EMBC.2019.8856387 31947227

[ref118] Secerbegovic A. (2016). Blood pressure estimation using video plethysmography faculty of electrical engineering, University of Tuzla, Bosnia and Herzegovina intervention center, Oslo university hospital, university of Oslo.

[ref119] Sereevoravitgul T. Kondo T. (2014). “A comparative study for heart rate measurement in video sequences.” in The International Conference on Information and Communication Technology for Embedded Systems (ICICTES2014). January 23 – 25, 2014; Ayutthaya, Thailand; 4.

[ref120] Shan L. Yu M. (2013). Video-based heart rate measurement using head motion tracking and ICA. *Proceedings of the 2013 6th International Congress on Image and Signal Processing, CISP 2013 1*, 160–164.

[ref121] Shao D. Liu C. Tsow F. Yang Y. Du Z. Iriya R.. (2016). Noncontact monitoring of blood oxygen saturation using camera and dual-wavelength imaging system. IEEE Trans. Biomed. Eng. 63, 1091–1098. doi: 10.1109/TBME.2015.2481896, PMID: 26415199

[ref122] Shao D. Yang Y. Liu C. Tsow F. Yu H. Tao N. (2014). Noncontact monitoring breathing pattern, exhalation flow rate and pulse transit time. IEEE Trans. Biomed. Eng. 61, 2760–2767. doi: 10.1109/TBME.2014.2327024, PMID: 25330153

[ref123] Sharma M. Barbosa K. Ho V. Griggs D. Ghirmai T. Krishnan S.. (2017). Cuff-less and continuous blood pressure monitoring: a methodological review. Secur. Technol. Des. 5:21. doi: 10.3390/technologies5020021

[ref124] Sharma V. K. Subramanian S. K. Arunachalam V. Rajendran R. (2015). Heart rate variability in adolescents – normative data stratified by sex and physical activity. J. Clin. Diagn. Res. 9, CC08–CC13. doi: 10.7860/JCDR/2015/15373.6662 PMC4625233 26557514

[ref125] Shirbani F. Hui N. Tan I. Butlin M. Avolio A. P. (2020). “Effect of ambient lighting and skin tone on estimation of heart rate and pulse transit time from video Plethysmography.” *Proceedings of the Annual International Conference of the IEEE Engineering in Medicine and Biology Society, EMBS*. July 20–24, 2020; Montreal, Canada (Virtual); 2642–2645. 10.1109/EMBC44109.2020.9176731 33018549

[ref126] Shoushan M. M. Reyes B. A. Rodriguez A. M. Chong J. W. (2021). Non-contact HR monitoring via smartphone and webcam during different respiratory maneuvers and body movements. IEEE J. Biomed. Health Inform. 25, 602–612. doi: 10.1109/JBHI.2020.2998399, PMID: 32750916

[ref127] Singh S. Rathee N. Gupta H. Zamboni P. Singh A. (2017). Contactless and hassle free real time heart rate measurement with facial video. J. Cardiac Critic. Care TSS 1, 24–29. doi: 10.1055/s-0037-1604202

[ref128] Song R. Zhang S. Cheng J. Li C. Chen X. (2020). New insights on super-high resolution for video-based heart rate estimation with a semi-blind source separation method. Comput. Biol. Med. 116:103535. doi: 10.1016/j.compbiomed.2019.103535, PMID: 31760272

[ref129] Sugita N. Obara K. Yoshizawa M. Abe M. Tanaka A. Homma N. (2015). Techniques for estimating blood pressure variation using video images. *Proceedings of the Annual International Conference of the IEEE Engineering in Medicine and Biology Society, EMBS*. November, 2015, 4218–4221. 10.1109/EMBC.2015.7319325 26737225

[ref130] Sun Y. (2011). Motion-compensated noncontact imaging photoplethysmography to monitor cardiorespiratory status during exercise. J. Biomed. Opt. 16:077010. doi: 10.1117/1.3602852, PMID: 21806290

[ref131] Sun Y. Hu S. Azorin-Peris V. Kalawsky R. Greenwald S. (2012). Noncontact imaging photoplethysmography to effectively access pulse rate variability. J. Biomed. Opt. 18:061205. doi: 10.1117/1.jbo.18.6.061205, PMID: 23111602

[ref132] Sun Y. Thakor N. (2016). Photoplethysmography revisited: From contact to noncontact, from point to imaging. IEEE Trans. Biomed. Eng. 63, 463–477. doi: 10.1109/TBME.2015.2476337, PMID: 26390439 PMC4822420

[ref133] Tan R. E. S. Lahiri A. (2020). Vascular anatomy of the hand in relation to flaps. Hand Clin. 36, 1–8. doi: 10.1016/j.hcl.2019.08.001, PMID: 31757342

[ref134] Tarassenko L. Villarroel M. Guazzi A. Jorge J. Clifton D. A. Pugh C. (2014). Non-contact video-based vital sign monitoring using ambient light and auto-regressive models. Physiol. Meas. 35, 807–831. doi: 10.1088/0967-3334/35/5/807, PMID: 24681430

[ref135] Tasli H. E. Gudi A. Uyl M. den (2014). Remote ppg based vital sign measurement using adaptive facial regions Vicarious Perception Technologies Intelligent Systems Lab Amsterdam, University of Amsterdam, The Netherlands. *International Conference on Image Processing (ICIP)*, 1410–1414.

[ref136] Urban S. Leitloff J. Hinz S. (2015). Improved wide-angle, fisheye and omnidirectional camera calibration. ISPRS J. Photogramm. Remote Sens. 108, 72–79. doi: 10.1016/j.isprsjprs.2015.06.005

[ref137] van der Kooij K. M. Naber M. (2019). An open-source remote heart rate imaging method with practical apparatus and algorithms. Behav. Res. Methods 51, 2106–2119. doi: 10.3758/s13428-019-01256-8, PMID: 31152386 PMC6797647

[ref138] van Gastel M. Stuijk S. de Haan G. (2016). Robust respiration detection from remote photoplethysmography. Biomed. Opt. Express 7:4941. doi: 10.1364/boe.7.004941 28018717 PMC5175543

[ref139] van Gastel M. Stuijk S. Overeem S. van Dijk J. P. van Gilst M. M. de Haan G. (2021). Camera-based vital signs monitoring during sleep - A proof of concept study. IEEE J. Biomed. Health Inform. 25, 1409–1418. doi: 10.1109/JBHI.2020.3045859, PMID: 33338025

[ref140] Verkruysse W. Svaasand L. O. Nelson J. S. (2008). Remote plethysmographic imaging using ambient light. Opt. Express 16:21434. doi: 10.1364/oe.16.021434 19104573 PMC2717852

[ref141] von Arx T. Tamura K. Yukiya O. Lozanoff S. (2018). The Face – A vascular perspective. A literature review. Swiss Dent. J. 128, 382–392. 29734800 10.61872/sdj-2018-05-405

[ref142] Wang W. den Brinker A. C. (2022). “Chapter 4 - Camera-based respiration monitoring: Motion and PPG-based measurement,” in Contactless Vital Signs Monitoring. eds. Wang W. Wang X. (London: Academic Press), 79–97.

[ref143] Wang W. den Brinker A. C. Stuijk S. de Haan G. (2017). Algorithmic principles of remote PPG. IEEE Trans. Biomed. Eng. 64, 1479–1491. doi: 10.1109/TBME.2016.2609282 28113245

[ref144] Wang C. Pun T. Chanel G. (2018). A comparative survey of methods for remote heart rate detection from frontal face videos. Front. Bioeng. Biotechnol. 6, 1–16. doi: 10.3389/fbioe.2018.00033 29765940 PMC5938474

[ref145] Wedekind D. Trumpp A. Gaetjen F. Rasche S. Matschke K. Malberg H.. (2017). Assessment of blind source separation techniques for video-based cardiac pulse extraction. J. Biomed. Opt. 22:035002. doi: 10.1117/1.jbo.22.3.035002, PMID: 28257535

[ref146] WHO (2021). Global strategy on digital health 2020–2025. Available at: https://www.who.int/docs/default-source/documents/gs4dhdaa2a9f352b0445bafbc79ca799dce4d.pdf (Accessed October 20, 2021).

[ref147] Wu H.-Y. Rubinstein M. Shih E. Guttag J. Durand F. Freeman W. (2012). Eulerian video magnification for revealing subtle changes in the world. ACM Trans. Graph. 31, 1–8. doi: 10.1145/2185520.2185561

[ref148] Yang Y. Lian C. Zhang H. Li L. Zhao Y. (2021a). Robust and remote measurement of heart rate based on a surveillance camera. bioRxiv [Epub ahead of print].

[ref149] Yang Z. Wang H. Lu F. (2021b). Assessment of deep learning-based heart rate estimation using remote Photoplethysmography under different illuminations. arXiv [Preprint].

[ref150] Yoon G. Lee J. Y. Jeon K. J. Park K.-K. Yeo H. S. Hwang H. T.. (2002). Multiple diagnosis based on photoplethysmography: hematocrit, SpO 2, pulse, and respiration. Optics Health Care Biomed. Optics: Diagnos. Treatment 4916:185. doi: 10.1117/12.482947

[ref151] Zaunseder S. Trumpp A. Wedekind D. Malberg H. (2018). Cardiovascular assessment by imaging photoplethysmography-a review. Biomed. Tech. 63, 617–634. doi: 10.1515/bmt-2017-0119 29897880

[ref152] Zhang Z. Member S. (2000). A Flexible New Technique.pdf. IEEE Trans. Pattern Anal. Mach. Intell. 22, 1330–1334. doi: 10.1109/34.888718

[ref153] Zhang Q. Wu Q. Zhou Y. Wu X. Ou Y. Zhou H. (2017). Webcam-based, non-contact, real-time measurement for the physiological parameters of drivers. Measurement: J. Int. Measur. Confed. 100, 311–321. doi: 10.1016/j.measurement.2017.01.007

[ref154] Zhang Q. Xu G. Q. Wang M. Zhou Y. Feng W. (2014). “Webcam based non-contact real-time monitoring for the physiological parameters of drivers.” in *4th Annual IEEE International Conference on Cyber Technology in Automation, Control and Intelligent Systems, IEEE-CYBER 2014*. June 4–7, 2014; Hong Kong; 648–652.

[ref155] Zhao F. Li M. Qian Y. Tsien J. Z. (2013). Remote measurements of heart and respiration rates for telemedicine. PLoS One 8:e71384. doi: 10.1371/journal.pone.0071384, PMID: 24115996 PMC3792902

[ref156] Zhao C. Mei P. Xu S. Li Y. Feng Y. (2019). “Performance evaluation of visual object detection and tracking algorithms used in remote photoplethysmography,” in *Proceedings - 2019 International Conference on Computer Vision Workshop, ICCVW 2019*, 1646–1655.

